# Methods to Assess Energy Expenditure of Resistance Exercise: A Systematic Scoping Review

**DOI:** 10.1007/s40279-024-02047-8

**Published:** 2024-06-19

**Authors:** Lachlan Mitchell, Luke Wilson, Grant Duthie, Kate Pumpa, Jonathon Weakley, Christopher Scott, Gary Slater

**Affiliations:** 1 School of Behavioural and Health Sciences, Australian Catholic University, North Sydney, Australia; 2 School of Behavioural and Health Sciences, Australian Catholic University, Strathfield, Australia; 3 Sports Performance, Recovery, Injury and New Technologies (SPRINT) Research Centre, Australian Catholic University, Melbourne, Australia; 4 Research Institute for Sport and Exercise, University of Canberra, Canberra, Australia; 5 School of Public Health, Physiotherapy and Sport Science, University College Dublin, Dublin, Ireland; 6 School of Behavioural and Health Sciences, Australian Catholic University, Brisbane, Australia; 7 Carnegie Applied Rugby Research (CARR) Centre, Carnegie School of Sport, Leeds Beckett University, Leeds, UK; 8 Department of Exercise, Health, and Sport Sciences, University of Southern Maine, Maine, USA; 9 School of Health, University of the Sunshine Coast, Sippy Downs, Australia

## Abstract

**Background:**

Nutrition guidance for athletes must consider a range of variables to effectively support individuals in meeting energy and nutrient needs. Resistance exercise is a widely adopted training method in athlete preparation and rehabilitation and therefore is one such variable that will influence nutrition guidance. Given its prominence, the capacity to meaningfully quantify resistance exercise energy expenditure will assist practitioners and researchers in providing nutrition guidance. However, the significant contribution of anaerobic metabolism makes quantifying energy expenditure of resistance exercise challenging.

**Objective:**

The aim of this scoping review was to investigate the methods used to assess resistance exercise energy expenditure.

**Methods:**

A literature search of Medline, SPORTDiscus, CINAHL and Web of Science identified studies that included an assessment of resistance exercise energy expenditure. Quality appraisal of included studies was performed using the Rosendal Scale.

**Results:**

A total of 19,867 studies were identified, with 166 included after screening. Methods to assess energy expenditure included indirect calorimetry (*n* = 136), blood lactate analysis (*n* = 25), wearable monitors (*n* = 31) and metabolic equivalents (*n* = 4). Post-exercise energy expenditure was measured in 76 studies. The reported energy expenditure values varied widely between studies.

**Conclusions:**

Indirect calorimetry is widely used to estimate energy expenditure. However, given its limitations in quantifying glycolytic contribution, indirect calorimetry during and immediately following exercise combined with measures of blood lactate are likely required to better quantify total energy expenditure. Due to the cumbersome equipment and technical expertise required, though, along with the physical restrictions the equipment places on participants performing particular resistance exercises, indirect calorimetry is likely impractical for use outside of the laboratory setting, where metabolic equivalents may be a more appropriate method.

**Supplementary Information:**

The online version contains supplementary material available at 10.1007/s40279-024-02047-8.

## Key Points

**Table:** 

Indirect calorimetry is used widely but is limited in quantifying the glycolytic cost of resistance exercise.
Indirect calorimetry measures during and immediately following resistance exercise in combination with blood lactate measures before and after exercise may be the most valid means of estimating total energy expenditure of resistance exercise. Metabolic equivalents may be the easiest tool to implement in the field.
Future research should aim to develop a more valid system to quantify the glycolytic contribution to resistance exercise and better understand how resistance exercise energy expenditure is currently estimated by practitioners in the field.

## Introduction

Resistance training can increase strength and power while reducing injury risk, and has been established as an essential auxiliary training tool in elite [1, 2], amateur [3], young [4], old [5], individual [6, 7] and team sport athletes [1, 2]. Furthermore, in sports such as bodybuilding, Olympic weightlifting, powerlifting and CrossFit, resistance exercise can be considered a primary mode of training [7–10]. Outside of a performance context, resistance exercise is used as an essential tool in athlete rehabilitation [11]. The often-periodised nature of competition preparation dictates variation in training characteristics based on the goals of each cycle. From a resistance exercise perspective, such variation would involve adjustments in training frequency (sessions per week), load (percentage of repetition maximum), volume (sets and repetitions) and movement velocity, amongst others. Such characteristics would therefore influence the overall workload experienced by the athlete, requiring a consideration of complementary facets of overall health and performance including nutrition.

The dietary requirements of athletes are primarily influenced by daily training load, but also impacted by other variables including health, injury status and environment [12, 13]. The variable nature of these factors within and across training phases and competition means sports dietitians must be dynamic with their nutrition support, providing customised direction around energy and nutrient requirements. Ultimately, energy intake requirements will be determined by the energy expended through resting metabolic rate and non-exercise activity energy expenditure, and, specifically in the context of the athlete, the hugely variable energy expenditure of exercise [14, 15]. As such, it is pertinent for sports dietitians to gather and assess athlete and training data to provide accurate guidance around dietary energy requirements for individual athletes. In particular, capturing estimates of exercise energy expenditure will support practitioners in providing this dietary energy guidance [12].

The reference method for quantifying total energy expenditure is direct calorimetry, but this is largely unavailable to researchers and practitioners [16]. Doubly labelled water, considered the reference measure for free living total energy expenditure, has been used to quantify the energy expenditure of various athlete cohorts [17–20]. However, this technique is limited in day-to-day practice due to costs and accessibility, while also failing to determine daily fluctuations in expenditure and lacking the capacity to quantify the energy expenditure of individual exercise sessions [15]. Physiological and metabolic complications that can result from imbalances in energy intake and the energy expenditure of exercise (low energy availability) include reductions in muscle protein synthesis and resting metabolic rate, as well as menstrual dysfunction, hormonal disruption and increased injury risk [21, 22]. Emerging evidence also suggests potential detrimental outcomes of acute imbalances between intake and expenditure within a day [23–25]. These physiological and metabolic complications demonstrate a clear justification for quantifying the energy expenditure of exercise [26]. As such, tools that allow this measurement are warranted. Given the prevalence of resistance exercise in athlete preparation, and by extension its contribution to exercise energy expenditure, the capacity to meaningfully quantify the energy expenditure of a resistance exercise session specifically will assist practitioners in providing energy requirement guidance. This will support athlete health and performance by optimising energy intake within and between days.

Indirect calorimetry is typically used to measure the energy cost of continuous, aerobic-based exercise. This technique relies on the subject achieving steady-state conditions and most of the energy being expended via aerobic energy pathways. Unlike aerobic exercise, though, resistance exercise depends on the significant contribution of anaerobic metabolism [27, 28]. Furthermore, the typically high-intensity, short-duration characteristics of a resistance exercise effort (i.e. a single set) means steady-state is not achieved [29]. As such, values of energy expenditure estimated through indirect calorimetry likely neglect a significant fraction of the energy cost of resistance exercise [29]. Depending on training variables, this anaerobic fraction may be over 40% of energy expenditure [30]. Attempts have been made to quantify the anaerobic contribution to resistance exercise energy expenditure. Blood lactate changes from pre- to post-exercise have been used to estimate the glycolytic energy cost [30, 31], with an energy equivalent used per unit increase in blood lactate concentration [32]. This method may be limited given that blood lactate is only an approximation of muscle blood lactate production. Indirect calorimetry during recovery periods between and immediately after exercise sets captures the energy supplied by the phosphagen system via measurement of the fast phase of excess oxygen uptake [33, 34]. Estimations of total energy expenditure have also been made on the basis of the Compendium of Physical Activities, with metabolic equivalent (MET) values of 3.5–6.0 used depending on the nature of the resistance exercise [35], while regression analyses have been performed to predict energy expenditure factoring in participant and training variables [36]. Both MET values and regression analyses are limited, though, by their dependence on indirect calorimetry to estimate energy expenditure, and resistance exercise MET values fail to account for disparity in energy expenditure depending on variability in muscle mass activation. Despite each of these methods being recognised, the range of techniques used to date suggests no accepted standard for quantifying the energy expenditure of resistance exercise. Further exploration of this field is therefore needed to establish recommendations for the accurate quantification of resistance exercise energy expenditure.

Factors within a resistance exercise session likely contribute to overall energy expenditure. The intensity of the exercise will influence the contribution of anaerobic systems to metabolic output [27], as will the proximity to failure, while movement velocity also influences energy expenditure [37]. Unlike continuous exercise, the high-intensity, intermittent nature of resistance exercise will result in significant energy expenditure during recovery periods (i.e. between sets and exercises) despite no external work being performed, largely attributed to the replenishment of high energy phosphates [28, 30]. The specific exercise itself also influences energy expenditure. Exercises activating larger amounts of muscle mass will inherently result in greater expenditure than those utilising smaller amounts of muscle mass, across a range of lifting intensities. For example, single sets of half squat and biceps curl performed to momentary failure at 80% one repetition maximum resulted in expenditures of 150.9 ± 20.9 and 35.8 ± 9.5 kJ/min, respectively [38]. The capacity to quantify total energy cost of a resistance exercise session will therefore require a system that considers these factors.

Given the prominent incorporation of resistance exercise in athlete programming, from both performance and rehabilitation perspectives, along with the importance of dietary guidance centred around individual energy and nutrient needs, accurately quantifying the energy cost of resistance exercise is warranted. Such a quantification would allow practitioners to better service athletes in meeting their dietary, health and performance goals. Researchers would also benefit through increasing the capacity to quantify the energy cost of training interventions, allowing a more accurate link between training variables and physiological outcomes. In addition, the capacity to quantify resistance exercise energy expenditure would also allow for a more accurate assessment of energy availability, given the critical role of quantifying energy expenditure of exercise. Therefore, the aim of this scoping review is to investigate the methods used to assess the energy expenditure of resistance exercise. This will support practitioners and researchers in estimating the energy cost of resistance exercise, and therefore enable more individual guidance around energy and nutrient needs.

## Methods

Reporting of this scoping review follows the Preferred Reporting Items for Systematic Reviews and Meta-Analyses (PRISMA) extension for scoping review guidelines. The protocol for this scoping review is registered at Open Science Framework (10.17605/OSF.IO/PUZWB).

### Eligibility Criteria

Experimental and observational study designs were eligible for inclusion. Review papers, abstracts and grey literature were not eligible. Studies not reported in English were excluded. Any study that included an assessment of the energy expenditure of resistance exercise was eligible for inclusion. This included single- and multi-set resistance exercise, circuit-based resistance exercise and exercise where body weight was used as resistance. Studies quantifying the energy expenditure of the usual training program of athletes, where the usual program included some form of resistance exercise, were also included, regardless of whether energy expenditure of the resistance training session itself was reported. No restrictions on participant age, sex, level of activity or health status were imposed.

### Search Strategy

The systematic search to identify studies was conducted by one researcher (L.M.) from the earliest record until 8 January 2024. Databases searched included Medline via Ovid, SPORTDiscus via EBSCOHost, CINAHL via EBSCOHost, and Web of Science. The search used key words and controlled vocabulary in the following combination: (“resistance train*” OR “resistance exercise*” OR “progressive resistance” OR “Weightlift*” OR “weight lift*” OR “Bodybuild*” OR “body build*” OR “weight train*” OR “strength train*” OR “progressive train*” OR “athlet*”) and (“energy expend*” OR “metabol*” OR “calorimet*” OR “energy availab*”). The full electronic search strategy is presented in Supplementary Fig. 1.

### Selection of Studies and Data Extraction

After the search was conducted and duplicates removed, manuscripts were screened by title and abstract by one researcher (L.M.). Full texts of all potentially eligible texts were independently screened by two researchers (L.M. and L.W.). Disagreements were resolved by discussion between screening authors. If a resolution could not be achieved, a third author (G.S.) made the final decision. All screening steps were conducted using Covidence systematic review software (Veritas Health Innovation, Melbourne, Australia).

A standardised Microsoft Excel spreadsheet (Microsoft Corporation, Redmond, WA, USA) was used for data extraction. All publications were extracted by L.M. Duplicate extraction was divided between L.W., G.D., K.P., J.W. and G.S. Extracted data included study characteristics (author, publication year, country and design), participant characteristics (sample size, age, sex and training status), resistance training details (exercises, sets, repetitions, load, rest and movement velocity), and outcome measures (energy expenditure assessment technique, energy expenditure values and post-exercise energy expenditure). A computer program (WebPlotDigitizer, Version 4.6) was used to calculate the mean and standard deviation of data reported in figures [39]. Final data extraction was crosschecked by L.M.

### Assessment of Reporting Quality

Although assessment of methodological quality is not required for scoping reviews, researchers deemed it appropriate for the overview of literature on the topic. The methodological quality of all included publications was assessed using the Rosendal Scale [40]. This scale assesses several factors associated with the minimisation of bias in areas such as participant selection, performance and data analysis. The scale is a combination of items from the Jadad scoring system [41], the Physiotherapy Evidence Database (PEDro) scale [42], and the Delphi List [43], in addition to recommendations contained in the CONSORT statement [44]. Scoring of each publication was determined by dividing the number of ‘yes’ responses by the number of relevant items, with excellent methodological quality indicated by a score of ≥ 60%. Each publication was scored by two researchers (L.M. and L.W.), with discrepancies resolved by discussion.

### Data Synthesis

A narrative approach to data synthesis was used. Characteristics of resistance exercise performed within included studies were categorised (single-exercise, multi-exercise, circuit training and group exercise). The technique used to estimate resistance exercise energy expenditure in each study was described, with studies grouped together on the basis of the assessment technique utilised. Due to variability in how energy expenditure was reported, the energy expenditure value within included studies was synthesised and presented as ranges. Tables and figures are used to summarise findings.

## Results

### Study Selection and Characteristics

**Fig. 1:**
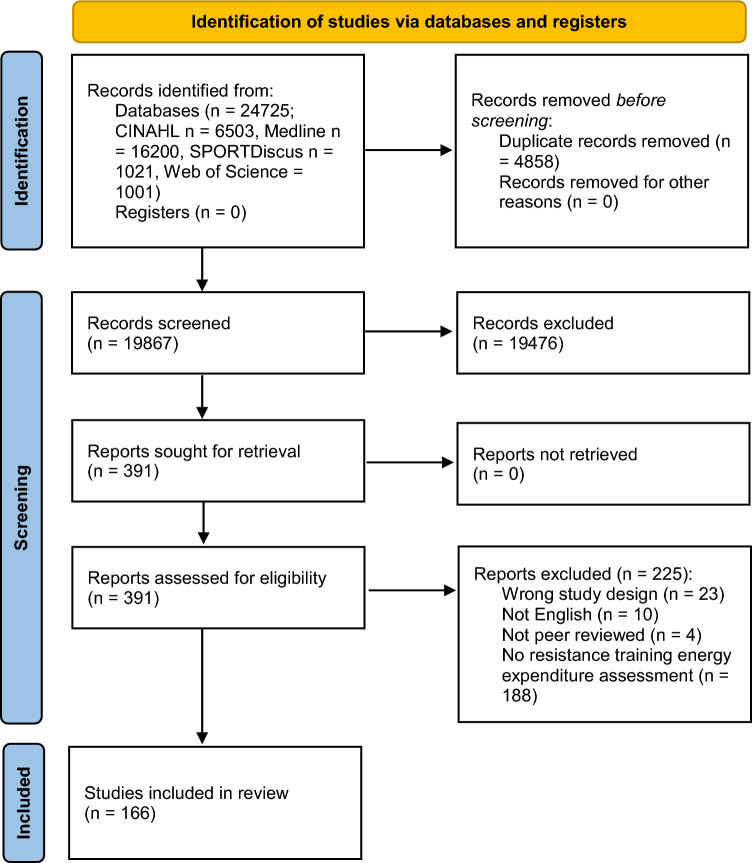
PRISMA flow diagram

The initial search yielded 24,725 citations. Following the removal of duplicates (*n* = 4858) and assessment of full text for eligibility (*n* = 391), a total of 166 articles met inclusion criteria [1, 14, 25, 27, 28, 30, 31, 33, 34, 36–38, 45–198]. Figure 1 outlines the flow of study identification. A total of 14 studies were published before 2000, 32 studies were published between 2000 and 2009, 89 studies were published between 2010 and 2019 and 31 studies were published between 2020 and 2023. By design, studies were cross-over studies (*n* = 84), cross-sectional studies (*n* = 39), repeated measures studies (*n* = 19), randomised controlled trials (*n* = 16), longitudinal observational studies (*n* = 4), single-group intervention studies (*n* = 2), a non-randomised controlled trial (*n* = 1) and a retrospective study (*n* = 1). Studies were conducted in the USA (*n* = 83), Europe (*n* = 34), South America (*n* = 18), Canada (*n* = 8), Australia (*n* = 6), Japan (*n* = 5), the UK (*n* = 6), New Zealand (*n* = 4), India (*n* = 1), and Singapore (*n* = 1).

The sample size of the studies ranged from 2 to 417. The mean age of participants ranged from 12.9 to 73.1 years. Six studies did not report mean participant age; however, two of these reported the age range of participants. Studies recruited trained participants (*n* = 99), recreationally active participants (*n* = 21), untrained participants (*n* = 23), clinical participants (*n* = 9) and a combination of trained and untrained participants (*n* = 4). Participant training status was not described in 10 studies.

### Resistance Training Characteristics

Details of the resistance training conducted in the included studies are presented in Supplementary Table 1. Of the 166 included studies, 42 used a single-exercise resistance training session, 60 used a multi-exercise resistance training session, 31 used a circuit training session, 5 measured group exercise classes, 2 studies used both single- and multi-exercise resistance training sessions and 26 studies had participants perform their usual resistance training program but did not describe the details of these programs.

### Energy Expenditure Assessment

**Fig. 2:**
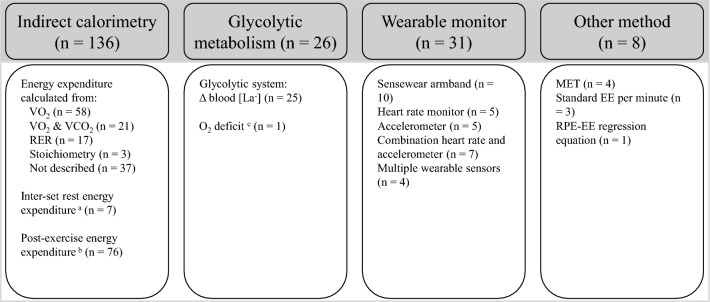
Methods of energy expenditure assessment reported in included studies. RER, respiratory exchange ratio; La^−^, lactate; MET, metabolic equivalents; EE, energy expenditure; RPE, rating of perceived exertion. ^a^Studies used a separate energy equivalent value for inter-set and inter-exercise rest periods to that used during exercise periods. ^b^Studies included a measure of energy expenditure in the period following resistance training. ^c^O_2_ deficit purported to capture both the glycolytic and phosphagen systems

The methods used to assess energy expenditure of resistance training are presented in Supplementary Table 1 and Fig. 2. Indirect calorimetry (*n* = 136) was the predominant method used to assess energy expenditure in included studies, along with blood lactate (*n* = 25) and wearable monitors (*n* = 13). Other methods reported were metabolic equivalents (MET) via the Compendium of Physical Activities (*n* = 4), a standard energy cost of resistance exercise per minute (*n* = 3), and an individual rating of perceived exertion–energy expenditure regression equation (*n* = 1).

#### Indirect Calorimetry

In total, 136 studies utilised indirect calorimetry to measure the energy expenditure of resistance exercise. These studies collected expired gas for either a portion or the entire duration of the resistance training. Whole room indirect calorimetry was used in 3 of 136 studies [94, 124, 125], with the remaining studies using either a metabolic cart (*n* = 131) or Douglas bags (*n* = 2). Most (*n* = 99) of the 136 studies defined how energy expenditure was calculated from indirect calorimetry. This calculation was based on a set energy equivalent per litre of oxygen (for example, 21.1 kJ/L O_2_; *n* = 58), an equation based on volume of oxygen (*V*O_2_) and volume of carbon dioxide (*V*CO_2_; for example, the Weir equation [199]; *n* = 21), an energy equivalent based on the respiratory exchange ratio (*n* = 17) or stoichiometric values (*n* = 3). The remaining 37 studies did not describe how energy expenditure was calculated from indirect calorimetry. A separate energy equivalent per litre of oxygen during inter-set and inter-exercise rest periods to that during the exercise period was used in seven studies.

#### Glycolytic Energy Expenditure

The glycolytic contribution to energy expenditure was estimated in 26 studies [27, 28, 30, 31, 33, 34, 37, 38, 47, 52, 99, 105, 107, 108, 117, 119, 121, 122, 141, 150, 154, 160, 168, 173, 174, 197]. Each of these studies included glycolytic measures in addition to indirect calorimetry measures. Blood lactate was measured in 25 of the 26 studies, with the change in blood lactate concentration (peak-rest [La^−^]) converted to an oxygen equivalent (3 mL O_2_/kg body mass/mmol [La^−^]), with energy expenditure calculated from this oxygen volume. One study used the accumulated oxygen deficit to quantify combined anaerobic energy expenditure [38].

#### Wearable Monitors

Wearable monitors were used in 31 studies to estimate energy expenditure of resistance exercise. This included 18 studies that measured total activity energy expenditure of athletes performing their usual training program that included resistance training. Monitors used were the Sensewear armband (*n* = 10) [1, 46, 51, 53, 71, 79, 80, 109, 157, 185], chest-mounted heart rate monitor (*n* = 5) [98, 117, 155, 156, 177], accelerometer (*n* = 5; wrist-mounted, *n* = 3; hip-mounted, *n* = 1; or waist-mounted, *n* = 1) [140, 147, 162, 163, 192], combination heart rate and accelerometer (*n* = 7; chest-mounted, *n* = 5; wrist- and chest-mounted, *n* = 1; or wrist-mounted, *n* = 1) [115, 129, 165, 167, 186, 190] and multiple wearable sensors (*n* = 4; wrist- and chest-mounted, *n* = 2; wrist-mounted, *n* = 1; and wrist-, hip- and waist-mounted, *n* = 1) [25, 57, 96, 131, 196]. Five of these studies were attempting to validate wearable monitors for measurement of energy expenditure against indirect calorimetry [51, 57, 96, 131, 157].

#### Post-exercise Energy Expenditure

Energy expenditure was measured in the period immediately post-exercise in 76 studies. Indirect calorimetry was used in each of these studies to assess post-exercise energy expenditure. Measurements continued for a pre-specified time frame (*n* = 57), ranging from 3 to 180 min, or until *V*O_2_ returned to a pre-determined value (*n* = 15) based on pre-exercise resting energy expenditure measurements or a defined O_2_ consumption (range: 4–5 ml O_2_/kg/min). In addition, resting metabolic rate was compared with pre-exercise metabolic rate at one or more of 12, 24, 36, 48, and 72 h after resistance training in four studies [45, 91, 101, 118].

### Resistance Training Energy Expenditure

Energy expenditure was reported as both an absolute expenditure (total kJ) and as a rate of expenditure (kJ/min). Energy expenditure values reported varied widely between studies. Absolute values ranged from 6–1575 kJ for single-exercise studies, 38–2957 kJ for multi-exercise studies, 235–1822 kJ for circuit exercise studies, and 1050–1402 kJ for group exercise studies. Relative expenditure values ranged from 8–151 kJ/min for single-exercise studies, 6–62 kJ/min for multi-exercise studies, 11–55 kJ/min for circuit exercise studies, and 4–6 kJ/min for group exercise studies. Reporting standard also varied, with studies reporting expenditure values as exercise alone, exercise plus inter-set rest, and exercise plus inter-set rest plus post-exercise recovery expenditure. Only a small number of studies (*n* = 34) accounted for resting metabolic rate within the reported energy expenditure values.

### Assessment of Reporting Quality

Results of the quality assessment are presented in Supplementary Table 2. Average Rosendal score was 66.7 ± 15.1%, with 118 of 166 studies achieving a score rated as excellent (≥ 60%).

## Discussion

This study aimed to review the methods used to assess resistance exercise energy expenditure. A total of 166 studies were included in the review, with the majority utilising indirect calorimetry to estimate energy expenditure. A smaller proportion of studies complemented indirect calorimetry by using methods to estimate the glycolytic contribution to energy expenditure. Wearable sensors were also utilised in included studies, many of which examined athletes performing their usual resistance training program as a component of a broader assessment of energy expenditure and quantifying energy availability. A range of resistance exercise sessions were examined in studies, including single and multi-exercise sessions, low and high intensity sessions, circuit training, and group exercise classes. Given the variability of metabolic characteristics of this range of exercises, the choice of method to quantify energy expenditure requires consideration of these characteristics. Although the majority of studies employed indirect calorimetry, there are limitations to this method pertinent to its application in resistance exercise.

### Indirect Calorimetry

Of the 166 studies included in this scoping review, 136 used indirect calorimetry to estimate energy expenditure. Although a valid method to measure the aerobic metabolic output during steady state exercise, indirect calorimetry is unable to quantify the glycolytic contribution to energy expenditure [168]. Resistance exercise is typically characterised by short duration, high intensity efforts. Loads of 80% of one repetition maximum or more are recommended for increasing muscular strength [200]. Exercises programmed to increase muscular power may vary in relative load, but movement velocity is emphasised [201]. Finally, while muscle hypertrophy can be achieved at a range of lifting intensities, proximity to failure is likely required [202]. This emphasis on high intensity, high velocity, fatigue-inducing exercise requires a significant anaerobic contribution to metabolic output. Therefore, to accurately quantify the energy expenditure of such exercise, employment of a system that can capture the anaerobic component accurately is necessary. Although indirect calorimetry is capable of accounting for the energy expended by the phosphagen system through the fast phase of oxygen uptake immediately after exercise [32], it is limited in measuring the anaerobic glycolytic component of expenditure. As such, indirect calorimetry alone is likely to underestimate the total energy expenditure of resistance exercise [203].

### Glycolytic Energy Expenditure

Given the limitations of indirect calorimetry for quantifying total expenditure, it is likely a requirement of any resistance exercise measure to include a method of gauging the glycolytic contribution to energy expenditure. Twenty-six of the reviewed studies used a measure of glycolytic energy expenditure in addition to indirect calorimetry. Specifically, blood lactate accumulation during the resistance exercise was measured to quantify glycolytic energy expenditure. The importance of including a measure of the glycolytic expenditure is reinforced when examining the contributions of aerobic and glycolytic systems during resistance exercise. For example, single sets of bench press performed to failure at 70% 1 repetition maximum showed a glycolytic expenditure of 26.5 ± 4.4 kJ and an aerobic expenditure of 4.9 ± 2.0 kJ. Similar differences in contribution were reported in loads ranging from 37 to 90% 1 repetition maximum [28]. Similarly, across two sets of bench press performed to failure at 70%, 80% or 90% 1 repetition maximum, the glycolytic pathway contributed a greater proportion of total energy expenditure compared to the aerobic pathway (32.9 ± 8.6 vs 14.2 ± 6.0 kJ, 33.1 ± 9.7 vs 8.9 ± 2.8 kJ, 21.5 ± 5.7 vs 6.2 ± 2.2 kJ, respectively) [27]. Sets of submaximal resistance exercise terminated prior to failure also showed significant contributions from glycolytic energy expenditure, albeit less than the aerobic contribution [47, 105]. In addition to these measures of glycolytic energy expenditure, previous studies have shown significant reductions in muscle glycogen content following resistance training, particularly in type II glycolytic muscle fibres, indicating a heavy reliance on glycolysis [204–207]. Clearly there is merit in including a measure of glycolytic expenditure when quantifying the energy cost of resistance exercise. Program design factors such as intensity and proximity to failure will likely influence the magnitude of the anaerobic system contribution to overall energy expenditure.

The accumulation of blood lactate, indicative of a positive balance between lactate production and clearance, represents an overall energy turnover greater than the rate of oxygen consumption. An energy equivalent value has been determined for each unit increase in blood lactate, established as 3 mL O_2_/kg body mass/mmol lactate, allowing the calculation of the glycolytic contribution to total expenditure [32]. Blood lactate accumulation can be measured using the difference in lactate pre- to post-exercise. A challenge though in the resistance exercise context is the intermittent effort, whereby relatively long duration rest intervals are typically interspersed amongst short duration sets. Because of this, measuring the change in blood lactate between each set may provide further insight into anaerobic expenditure than the pre- and post-exercise difference alone. This serves to highlight the nuanced approach required to quantify resistance exercise energy expenditure, where consideration of training specific characteristics is necessary. A degree of pragmatism is also necessary; although further insight may be gained from inter-set measures, the intrusive nature of blood lactate measurement needs to be recognised, particularly outside the laboratory setting. For this reason, a lactate monitoring system akin to continuous glucose monitoring may provide additional insight. The use of blood lactate measures is not itself without limitations though. One such limitation is that blood lactate concentration at best provides only an approximate description of muscle lactate levels and glycolytic ATP resynthesis [203]. Further research elucidating a more valid measure of glycolytic energy expenditure is therefore required. The influence of muscle fibre composition of athletes on glycolytic energy expenditure during resistance exercise also warrants investigation based on the differing metabolic capacities of muscle fibre types [208].

### Wearable Monitors

Wearable monitors were utilised in 31 included studies, with the Sensewear armband the most frequently used (*n* = 10). Of these 28 studies, 18 estimated total energy expenditure of participants conducting their usual training routine that included resistance exercise. While accessibility and ease of application make wearable technologies an attractive tool for monitoring energy expenditure, such tools have shown limited capacity to provide an accurate quantification of resistance exercise expenditure [57]. Five included studies attempted to validate wearable devices against indirect calorimetry [51, 57, 96, 131, 157]. Resistance exercise protocols included body weight exercises, circuit resistance exercise, and traditional resistance exercise. The devices used included wrist-, chest-, waist-, hip-, and arm-worn, such as the Sensewear armband. Overall findings varied, but four of the five studies found either poor correlation or significant differences in absolute energy expenditure values between monitors and indirect calorimetry [51, 57, 96, 131]. Monitors were found to both under- and overestimate energy expenditure values against indirect calorimetry, with mean absolute percentage errors ranging markedly (15.1–57.0%) [51, 57, 96, 131], and similar marked variance in correlations (*r* = 0.02–0.74) [57, 96, 131]. One study found the Sensewear armband Mini and BodyMedia FIT had very large correlations with indirect calorimetry (*r* = 0.77–0.78) and a trivial to small percent mean change from indirect calorimetry during traditional resistance training [157]. It is notable though that none of these validation studies included a measure of glycolytic expenditure with indirect calorimetry, meaning the criterion measures used in these studies may themselves be limited. In addition, the location of devices on the body may influence validity, with suggestions that arm-worn devices may have limited capacity to capture lower extremity exercise and vice versa [157]. Given the limited validity currently available for wearable devices in a resistance training context, the use of such devices should proceed with caution when quantifying energy expenditure, particularly with commercially available devices which show significant variability [131].

### Post-exercise Energy Expenditure

Resistance exercise consistently demonstrates an elevation in energy expenditure beyond the acute exercise period. This is evidenced by an increased heart rate and *V*O_2_ relative to pre-exercise for at least 30 min after the cessation of lifting [55, 149, 151, 161]. Four of the reviewed studies included a measure of metabolic rate at one or more of 12, 24, 36, 48, and 72 h after resistance exercise. Results in these studies varied, with metabolic rate remaining elevated for up to 12–72 h [101, 118], although these longer-term elevations are likely trivial, and in part attributable to exercise-induced muscle damage [209] along with measurement noise [210]. Contrary to this is the acute elevation in *V*O_2_ and energy expenditure immediately following a resistance exercise effort relative to pre-exercise. The *V*O_2_ measured in the first minute following an exercise set has been observed to increase beyond values measured during the set [151]. This increase in *V*O_2_ in the minutes following a resistance effort is considered the fast phase of oxygen kinetics and accounts for the energy expenditure of the phosphagen system. The absolute contribution of this measure may be equivalent to, if not greater than, the contribution from either aerobic metabolism or glycolytic metabolism during the exercise set [27, 31]. Acknowledging the dependence on anaerobic energy systems during resistance exercise and the observable metabolic output immediately following a resistance exercise effort related to the phosphagen system, it appears pertinent to include measures of post-exercise oxygen consumption in any calorimetric estimation of resistance exercise energy expenditure. These measures should include rest periods between sets and the recovery period following the final set. It is suggested that recovery measures continue until *V*O_2_ returns to 5 mL/kg/min (a typical standing oxygen uptake) [28].

### Metabolic Equivalents

An underutilised approach to estimating energy expenditure in the reviewed studies was the use of MET values. Four studies quantified the energy expenditure of resistance exercise using MET [14, 68, 132, 194]. Each study assessed total energy expenditure of participants during their regular training and competition routines, which included resistance exercise. Resistance exercise was assigned a MET value according to the Compendium of Physical Activities [35], from which energy expenditure was calculated. A strength of the MET system is its simplicity of application in estimating energy expenditure. No equipment is necessary, with users assigning an appropriate value based on training characteristics before undertaking basic calculations to convert MET into an energy expenditure. For this reason, its use is appealing for practitioners in the field. A challenge, though, in the resistance training context is selecting an appropriate MET value to assign. The most recent iteration of the Compendium provides MET values of 3.5, 5.0 and 6.0 for resistance exercises on the basis of the type and intensity of training, derived from published literature [35]. Previous research has measured MET values during resistance exercise and compared these with the Compendium of Physical Activities. Depending on population and training characteristics, measured values range between 3.0 and 8.0 MET, with the higher values observed in circuit style training characterised by high repetitions with minimal rest periods [144, 145, 183, 211]. Similar variability was identified in the energy expenditure measured in studies included in the current review, suggesting a range of equivalent MET values. The almost twofold difference between highest and lowest MET values from the Compendium of Physical Activities, and the large variability in energy expenditure values of reviewed studies, highlights the importance of clearly defining resistance exercise and considering the amount of muscle mass activated during exercise when using the MET system. In addition to the challenge of selecting the most relevant MET value, this system may also underestimate energy expenditure of resistance exercise on the basis of how the values have been derived. The MET values are obtained from measurements of the oxygen cost of the activity, thus neglecting the meaningful contribution to expenditure from glycolysis. Furthermore, Compendium instructions recommend applying the MET value to the active exercise period and excluding periods of rest between sets [35]. This process would likely then miss the substantial energy expenditure associated with the phosphagen system. At minimum though, the MET system may be applied in the field to provide an estimate of approximate energy expenditure. Consideration should also be given to the health status of the individual. A modified MET value may be more suitable for athletes presenting with anomalies relating to muscle activation, including some paralympic athletes, given that the resistance exercise MET value for wheelchair users is 2.2 [212].

### Limitations

This scoping review has limitations which should be considered. Many studies did not define how energy expenditure was calculated from expired gas analysis, and few studies reported the reproducibility of energy expenditure measures. The variability in units used to report energy expenditure (absolute expenditure and rate of expenditure) limited the capacity to report the values identified in reviewed studies. The energy expenditure of resistance exercise may vary on the basis of training age and training goals. Due to the inconsistencies in energy expenditure reporting, the influence of these variables was unable to be examined.

## Conclusion

The capacity to quantify the energy expenditure of resistance exercise would allow researchers and practitioners to better address the energy and nutrient needs of athletes. Most included studies in this scoping review used indirect calorimetry to estimate energy expenditure during resistance exercise. This technique captures the aerobic energy expenditure, with oxygen uptake measured in the minutes immediately following exercise also estimating the energy expenditure of the phosphagen system. A small number of studies included blood lactate analysis before and after exercise to account for the glycolytic contribution to expenditure. Wearable monitors and the MET system were also used to estimate energy expenditure in a small number of studies.

Based on these findings, it is suggested that laboratory measures of resistance exercise energy expenditure utilise blood lactate analysis to capture the glycolytic system in addition to indirect calorimetry measured during and in the minutes immediately following exercise to account for the aerobic and phosphagen systems. Measures of energy expenditure in the field will likely rely largely on estimations. The MET system may be viable despite having limitations, whereas wearable monitors should be used with caution based on current validity measures. The use of multiple devices, capturing both upper and lower body as well as variable sensor inputs, may provide more valid estimates of resistance exercise energy expenditure. Additional evidence is required to validate such a method.

There are several questions future research could investigate. Identifying other means to quantify the glycolytic contribution to expenditure beyond change in blood lactate would be valuable. Investigating the influence of muscle fibre composition on glycolytic energy expenditure would also be of value based on differences in substrate utilisation between fibre types. Developing a compendium of resistance exercises and their approximate energy expenditure values may assist practitioners. Currently, there is a paucity of literature examining energy expenditure in paralympic athletes. Future research in this population is warranted to confirm transferability of the measurement techniques identified in this review. Finally, understanding the methods currently used by practitioners in the field to estimate energy expenditure of resistance exercise would support the findings of the present scoping review.

## Supplementary Information

Below is the link to the electronic supplementary material.

Supplementary file1 (PDF 669 KB)

## Declarations

### Availability of data and material

All data and material in this manuscript are from peer-reviewed publications.
