# Supplementary material for: Methods to Assess Energy Expenditure of Resistance Exercise: A Systematic Scoping Review
Source: Sports Med. 2024 Jun 19;54(9):2357–72. doi: 10.1007/s40279-024-02047-8 (PMC11393209; doi:10.1007/s40279-024-02047-8)
Supplement: Supplementary file 1 — Supplementary file1 (PDF 669 KB) [file 40279_2024_2047_MOESM1_ESM.pdf]

Methods to assess energy expenditure of resistance exercise: A systematic scoping review

Journal: Sports Medicine

Lachlan Mitchell[1], Luke Wilson[1], Grant Duthie[2,3], Kate Pumpa[4,5], Jonathon Weakley[3,6,7], Christopher Scott[8], Gary Slater[9]

1. School of Behavioural and Health Sciences, Australian Catholic University, North Sydney, Australia
2. School of Behavioural and Health Sciences, Australian Catholic University, Strathfield, Australia
3. Sports Performance, Recovery, Injury and New Technologies (SPRINT) Research Centre, Australian Catholic University
4. Research Institute for Sport and Exercise, University of Canberra, Canberra, Australia
5. School of Public Health, Physiotherapy and Sport Science, University College Dublin, Ireland
6. School of Behavioural and Health Sciences, Australian Catholic University, Brisbane, Australia
7. Carnegie Applied Rugby Research (CARR) Centre, Carnegie School of Sport, Leeds Beckett University, Leeds, United Kingdom
8. Department of Exercise, Health, and Sport Sciences, University of Southern Maine, Maine, United States
9. School of Health, University of the Sunshine Coast, Sippy Downs, Australia

Corresponding author: Lachlan Mitchell, lachlan.mitchell@acu.edu.au

Supplementary Figure 1. Complete search strategy

Medline via Ovid<br>1: “resistance train*”<br>2: “resistance exercise*”<br>3: “progressive resistance”<br>4: Weightlift*<br>5: “weight lift*”<br>6: Bodybuild*<br>7: “body build*”<br>8: “weight train*”<br>9: “strength train*”<br>10: “progressive train*”<br>11: athlet*<br>12: Combine 1-11 with OR<br>13: “energy expend*”<br>14: metabol*<br>15: calorimet*<br>16: “energy availab*”<br>17: combine 13-16 with OR<br>18: 12 AND 17
SportDiscus via EBSCOHost<br>1: “resistance train*”<br>2: “resistance exercise*”<br>3: “progressive resistance”<br>4: Weightlift*<br>5: “weight lift*”<br>6: Bodybuild*<br>7: “body build*”<br>8: “weight train*”<br>9: “strength train*”<br>10: “progressive train*”<br>11: athlet*<br>12: “energy expend*”<br>13: metabol*<br>14: calorimet*<br>15: 1 OR 2 OR 3 OR 4 OR 5 OR 6 OR 7 OR 8 OR 9 OR 10 OR 11

16: “energy availab*”<br>17: 12 OR 13 OR 14 OR 16<br>18: 15 AND 17
Cinahl via EBSCOhost<br>“resistance train*” OR “resistance exercise*” OR “progressive resistance” OR Weightlift* OR “weight lift*” OR Bodybuild* OR “body build*” OR “weight train*” OR “strength train*” OR “progressive train*” OR athlet* AND “energy expend*” OR metabol* OR calorimet* OR “energy availab*”
Web of Science<br>1: “resistance train*” OR “resistance exercise*” OR “progressive resistance” OR Weightlift* OR “weight lift*” OR Bodybuild* OR “body build*” OR “weight train*” OR “strength train*” OR “progressive train*” OR athlet* (Keyword Plus)<br>2: “energy expend*” OR metabol* OR calorimet* (Keyword Plus)<br>3: 1 AND 2

Supplementary Table 1. Characteristics and energy expenditure assessment methods of included studies.

Study	N (sex)	Age	Training status	Resistance training	Energy expenditure technique
Abboud, G. J. et al (2013) [45]	8 (8 male)	22 ± 3	Resistance trained	2 sessions, varying by total load lifted. 4 exercises per session. 6-8 repetitions per set at 85% 1RM. Session 1 volume load 10000kg, session 2 volume load 20000kg	Indirect calorimetry
Aerenhouts, D. et al (2011) [46]	16 (6 girls)	16.5 ± 1.6	Athlete	Participants performed their usual resistance training program. Program not described.	Sensewear armband
Aniceto, R. R. et al (2013) [47]	10 (10 male)	21.3 ± 3.3	Resistance trained	Session1: Traditional resistance training, 8 exercises, 3 sets per exercise (24 sets total), 10 repetitions per set at 60% 1RM<br>Session 2: Circuit training, 3 rounds, 8 stations (24 stations total), 10 repetitions per station at 60% 1RM	Indirect calorimetry. Exercise EE (kJ) = 21.1 kJ/L O_2; rest EE: 19.6 kJ/L O_2<br>Δ blood $[La^-]$. 3 mL O_2 x mass (kg) x $\Delta[La^-]$ (mmol); 21.1 kJ/L O_2<br>Total EE = aerobic EE + lactate EE
Anthierens, A. et al (2019)* [48]	30 (30 male)	Cyclists: 20.3 ± 0.7<br>Judokas: 19.7 ± 0.4<br>Untrained: 23.1 ± 0.4	Trained and untrained	Single exercise. 5-minutes continuous trunk extension at 70% maximal work	Indirect calorimetry. EE (J/s) = [(3.869 x VO_2) + (1.195 x VCO_2) x (4.186 / 60)] x 1000
Barreto, A. C. et al (2010) [49]	20 (20 female)	34.6 ± 5.5	Resistance trained	2 sessions varying by repetition velocity. Circuit format. 6 exercises, 3 sets of 10 repetitions at 70% 1RM. Session 1 - 1s concentric, 1s eccentric; session 2 - 2s eccentric, 2s concentric	Indirect calorimetry
Beckham, S. G. & Earnest, C. P. (2000) [50]	30 (18 female)	Females: 25.7 ± 7.2<br>Male: 24.2 ± 5.7	Trained and untrained	2 group exercise classes, 14-minute duration, using bar and dumbbells. Classes varied by resistance (light or moderate).	Indirect calorimetry. Exercise EE calculated using RER-based kcal/min
Benito, P. J. et al (2016) [52]	29 (14 female)	Females: 20.7 ± 3.4<br>Male: 22.5 ± 2.6	Resistance trained	3 circuit sessions: machine weights; free weight; combination machine and free weights with treadmill running. 3 rounds, 8 stations, 15 reps per station at 70% 15RM	Indirect calorimetry. Exercise EE (kcal) = 5 kcal/L O_2<br>Δ blood $[La^-]$. 3 mL O_2 x mass (kg) x $\Delta[La^-]$ (mmol)<br>Total EE = aerobic EE + lactate EE
Benito, P.; et al (2012) [51]	29 (17 female)	Females: 21.5 ± 2.6<br>Male: 24.2 ± 1.9	Resistance trained	3 circuit resistance training sessions, varying by intensity. 3 rounds, 8 stations per round at either 30%, 50%, or 70% 15RM	Indirect calorimetry. Exercise EE (kcal) = 5 kcal/L O_2<br>Sensewear armband
Berthiaume, M. P. et al. (2015) [53]	40 (20 female)	Female: 32.2 ± 6.3<br>Male: 31.3 ± 5.4	Resistance trained	Body pump class, 60-minute duration	Sensewear armband
Bertucci, W. M. et al (2015) [54]	9 (9 male)	24.0 ± 3.0	Resistance trained	Squat exercise, single set of 3-minute duration, 60 repetitions per set, with external load of 50% body weight, performed on vibrating platform under 3 conditions: No vibration; vibrating at 40 Hz, 2 mm amplitude; vibrating at 40 Hz, 4 mm amplitude.	Indirect calorimetry. Exercise EE (J/s) = (3.869 x VO_2 + 1.195 x VCO_2) x (4.186/60) x 1000
Binzen, C. A. et al (2001) [55]	10 (10 female)	29.2 ± 3.0	Resistance trained	Single resistance training session. 10 exercises, 3 sets per exercise, 10 repetitions per set at 70% 1RM. 1 min rest between sets	Indirect calorimetry. Exercise EE based on VO_2 and RER values.
Bloomer, R. J. (2005) [56]	10 (0 female)	24.3 ± 3.8	Resistance trained	Squat exercise session, 30-minute duration, intermittent sets performed to failure at 70% 1RM	Indirect calorimetry. Exercise EE (kcal) = 5.05 kcal/L O_2

				(usually 5-12 repetitions), 90-120 seconds rest between sets	
Boudreaux, B. D. et al (2018) [57]	50 (28 female)	Female: 22.7 ± 3.0<br>Male: 22.0 ± 2.7	Recreationally trained	Single circuit resistance training session. 3 rounds, 4 exercises, 10 repetitions per exercise at 10RM	Indirect calorimetry<br>7 different wearable sensors (Apple Watch series 2, Fitbit Blaze, Fitbit Charge 2, Garmin Viviosmart HR, TomTom Touch, Polar A360, Polar H7)
Bradley, W. J. et al (2015) [1]	44 (44 male)	Forwards: 28.0 ± 2.8<br>Backs: 25.1 ± 3.8	Athlete	Participants performed their usual resistance training program. Program not described.	Sensewear armband
Brentano, M. A. et al (2016) [58]	20 (20 male).	Grouped session: 26.6 ± 3.4<br>Separated session: 24.9 ± 2.6	Recreationally trained	2 resistance training sessions, varying by exercise pairings. Grouped session paired supersets using exercises of the same muscle group (pec deck and bench press, leg press and leg extension); separated session paired supersets using exercises of different muscle groups (bench press and leg extension, leg press and pec deck). 5 sets, 8-10 repetitions per set at 85% 1RM	Indirect calorimetry. Exercise EE (kcal) = 4.82 kcal/L O_2
Brewer, C. B. et al (2021) [59]	9 (9 male)	22.1 ± 1.9	Resistance trained	Single circuit resistance training session. 3 rounds, 9 exercises, 15 repetitions at 40% 1RM	Indirect calorimetry. Exercise EE (kcal/min) = 3.781 x VO_2 + 1.237 x VCO_2
Brisebois, M. F et al. (2022) [60]	30 (15 female)	Female: 30.0 ± 5.0<br>Male: 26.0 ± 7.0	Recreationally trained	2 resistance training sessions. Traditional training used 3 exercises (back squat, leg extension, calf raise), 1-3 sets, 10-20 repetitions per set at 50% 1RM, followed by 20 minutes treadmill running. Functional training used 1 exercise (back squats), 5 sets, 5 repetitions per set at 75% 1RM, followed by 15 minutes circuit training.	Indirect calorimetry
Brunelli, D. T. et al (2019) [34]	11 (11 male)	22.0 ± 3.0	Untrained	2 leg extension sessions, varying by intensity. 3 sets performed to failure at either 30% 1RM or 80% 1RM.	Indirect calorimetry.<br>Δ blood $[La^-]$. 3 mL O_2 x mass (kg) x $\Delta[La^-]$ (mmol)<br>Anaerobic alactic energy estimated using an exponential model to fit the initial 7 minutes from VO_2 recovery period, considered the post-exercise fast VO_2 kinetics.<br>Total EE = aerobic EE + lactate EE + alactic EE
Cadieux, S. et al. (2014) [61]	16 (8 female)	Female: 22.8 ± 3.0<br>Male: 21.1 ± 2.0	Untrained	Single resistance training session, continued until a predetermined energy expenditure achieved (4 kcal/kg). Exercises performed as pairs, 4 sets, 10 repetitions per set at 70% 1RM.	Indirect calorimetry.
Caruso, J. F. et al. (2012)* [62]	28 (28 female)	20.1 ± 0.2	Athlete and sedentary	Tonic and phasic exercise sessions performed on a non-gravity-dependent resistance training machine. Both sessions were 2 sets, 1 minute per set, performed with maximal effort.	Indirect calorimetry. Exercise EE = 5 kcal/L O_2
Caruso, J. F. et al. (2006) [63]	14 (7 females)	Not reported	Not described	Single set resistance training session using an isoinertial leg press machine, performed on 3 occasions. 8 repetitions, performed with maximal effort.	Indirect calorimetry. Exercise EE = 5 kcal/L O_2
Caruso, J. F. & Hernandez, D. A. (2002)* [64]	23 (12 female)	22.7 ± 0.9	Not described	Single exercise using an isoinertial leg press machine, performed on 3 occasions. 3 sets, 8 repetitions per set, performed with maximal effort.	Indirect calorimetry. Exercise EE = 5 kcal/L O_2

Caruso, J. F. et al. (2003)* [65]	34 (25 female)	Female: 22.5 ± 1.1<br>Male: 23.5 ± 1.9	Not described	Single exercise resistance training using an isoinertial leg press machine, using 2 separate protocols each performed twice, varying by repetition type (concentric only contractions, concentric-eccentric contractions). 3 sets, 8 repetitions per set, performed with maximal effort.	Indirect calorimetry. Exercise EE = 5 kcal/L O_2
Caruso, J. F. et al. (2005) [66]	31 (18 female)	Not reported	Not described	Single exercise resistance training session using an isoinertial calf press machine, performed on 3 occasions. 3 sets, 10 repetitions per set, performed with maximal effort.	Indirect calorimetry. Exercise EE = 5 kcal/L O_2
Chatzinikolaou, A. et al. (2008) [67]	17 (17 male); 9 lean, 8 obese	Lean: 23.8 ± 1.2<br>Obese: 23.4 ± 0.8	Not described	Single circuit resistance training session. 3 rounds, 10 exercises, 10-12 repetitions per exercise at 70-75% 1RM.	Indirect calorimetry
Cherian, K. S. et al. (2018) [68]	40 (19 girls)	Boys: 11.7 ± 2.1<br>Girls: 12.2 ± 1.8	Athlete	Program not described.	Indirect calorimetry, MET values from compendium of activities
Correa, C. S. et al. (2015) [69]	36 (36 female)	58.9 ± 5.8	Untrained	2 resistance training groups, varying by volume. High volume: 8 exercises, 3 sets, 15 repetitions per set at 65% 1RM; low volume: 8 exercises, 1 set, 15 repetitions per set at 65% 1RM.	Indirect calorimetry. Exercise EE = 5 kcal/L O_2
Correa, C. S. et al. (2014) [70]	39 (39 female)	59.5 ± 4.8	Untrained	2 resistance training sessions, varying by volume. High volume: 8 exercises, 3 sets, 15 repetitions per set at 15RM; Low volume: 8 exercises, 1 set, 15 repetitions per set at 15RM.	Indirect calorimetry. Exercise EE = 5.05 kcal/L O_2
Costello, N. et al. (2019) [71]	8 (8 male)	17.0 ± 1.0	Athlete	Participants performed their usual resistance training program. Program not described.	Sensewear armband
Crommett, A. D. & Kinzey, S. J. (2004) [72]	17 (17 female); 10 lean, 7 obese	Lean: 19.5 ± 0.5<br>Obese: 20.4 ± 0.5	Untrained	Single resistance training session. 5 exercises, 3 sets, 8-12 repetitions pe set at 70% 10RM.	Indirect calorimetry. Exercise EE = 5 kcal/L O_2
Cunha, F. A. et al. (2019) [73]	9 (4 females)	70.3 ± 4.8	Recreationally trained	Single circuit resistance and aerobic training session. Outdoor gym equipment utilising body weight as resistance. Each resistance training exercise was either 1 or 2 sets, 15 repetitions per set.	Indirect calorimetry. Exercise EE = (3.941 x VO_2 + 1.106 x VCO_2)
Curtis, C. et al. (2023) [192]	12 (12 female)	26.0 ± 4.0	Athlete	Participants performed their usual resistance training program. Program not described.	Wearable triaxial accelerometer (Actigraph)
Da Silva, M. E. et al. (2007) [74]	17 (17 male)	18.3 ± 0.2	Resistance trained	2 half squat sessions, performed with or without whole body vibration. 3 sets, 15 repetitions per set at 95% 10RM.	Indirect calorimetry
Danoff, P. L. & Danoff, J. V. (1982) [75]	5 (5 female)	24.0	Not described	2 leg press sessions, varying by contraction type. Static (isometric): 5 minutes of continuous contractions using loads of 23kg, 27kg, 32kg, 36kg; Dynamic (isotonic): 5 minutes of continuous concentric/eccentric contractions, 20 repetitions per minute using loads of 14kg, 18kg, 23kg, 27kg.	Indirect calorimetry. Exercise EE = 5.05 kcal/L O_2
DeGroot, D. W. et al. (1998) [77]	9 (9 male)	63.3 ± 7.5	Clinical	4 circuit resistance training sessions, varying by recovery time and load. 3 rounds, 6 exercises, 30 seconds at each exercise, 40 or 60% 1RM, 30 or 60s rest between exercises.	Indirect calorimetry. Exercise EE = 5.05 kcal/L O_2

Del-Cuerpo, I. et al. (2023) [193]	29 (16 female)	24.9 ± 4.6	Resistance trained	2 half squat sessions, performed twice, varying by load and repetitions. 3 sets, 50 or 75% 1RM, 30 or 12 repetitions, 5 minutes rest.	Indirect calorimetry
De Looze, M. P. et al. (1994)# [76]	6 (6 male)	20-26	Untrained	4 deadlift sessions, varying by contraction type. Concentric-eccentric, eccentric only, concentric only, “simulated lifting and lowering” (no external load). 5 minutes of continuous lifting at 3.5kg, 7.5kg, 11.4kg, 15.4kg (for concentric-eccentric, concentric, eccentric), 18.4kg, 23.7kg (eccentric)	Indirect calorimetry
Dos Santos, T. R. et al. (2022) [78]	7 (3 female)	58.0 ± 12	Clinical	Circuit resistance training session performed twice. 3 rounds, 10 exercises, 15 repetitions at 100% 15RM.	Indirect calorimetry
Drenowatz, C. et al. (2015)a [79]	9 (9 male)	27.0 ± 2.3	Untrained	Single resistance training session performed 3 times per week for 16 weeks. 10 exercises, 3 sets, 8-12 repetitions per set.	Sensewear armband
Drenowatz, C. et al. (2015)b [80]	417 (213 female)	27.7 ± 3.8	Not described	Self-selected resistance training program	Sensewear armband
Dudley, G. A. et al. (1991) [81]	17 (17 male); 9 concentric-eccentric, 8 8oncentric only	Concentric-eccentric: 32.0 ± 3.0<br>Concentric only: 33.0 ± 3.0	Resistance trained	2 leg press sessions varying by contraction type. 4 sets, at a load selected to induce failure within the 7-10 repetition range. Concentric-eccentric contractions, concentric only contractions.	Indirect calorimetry. Exercise EE = 5 kcal/L O_2
Egger, T. & Flueck, J. L. (2020) [82]	14 (6 female)	Female: 33.8 ± 12.1<br>Male: 35.8 ± 7.6	Athlete	Participants performed their usual resistance training program. Program not described.	Estimated using a standard expenditure per minute. Exercise EE = 2.2-2.3 kcal/kg/minute of resistance exercise.
Falcone, P. H. et al. (2015) [83]	9 (9 male)	25.0 ± 7.0	Recreationally trained	Resistance training session: 6 exercises, 3 sets, 10 repetitions per set at 75% 1RM.<br>High intensity interval training session on hydraulic resistance system: 8 exercises, 4 sets, 20s per set	Indirect calorimetry. Expenditure of fat and carbohydrate determined. Exercise EE calculated as 4 kcal/g carbohydrate, 9 kcal/g fat.
Farinatti, P. T. & Castinheiras Neto, A. G. (2011) [84]	10 (10 male)	26.0 ± 3.0	Resistance trained	4 single exercise sessions, varying by exercise (leg press or chest fly) and rest interval (1-minute or 3-minutes). 5 sets, 10 repetitions at 15RM.	Indirect calorimetry. Exercise EE = 5.05 kcal/L O_2
Farinatti, P. T. et al (2009) [85]	10 (10 female)	22.0 ± 2.0	Resistance trained	2 sessions varying by exercise order. 3 exercises, 3 sets to failure at 10RM.	Indirect calorimetry. Exercise EE = 5.05 kcal/L O_2
Fatouros, I. G.; et al. (2005) [86]	50 (50 male)	Control: 69.8 ± 5.1<br>Low intensity: 71.1 ± 3.6<br>Moderate intensity 69.7 ± 3.8<br>High intensity: 70.8 ± 2.8	Untrained	3 exercise groups varying by intensity and repetitions. 10 exercises, 3 sets. Low intensity: 45-50% 1RM; moderate intensity: 60-65% 1RM; high intensity: 80-85% 1RM.	Indirect calorimetry. Exercise EE = 5.05 kcal/L O_2
Ferrari, R. et al. (2018) [87]	11 (11 female)	22.4 ± 2.3	Resistance trained	4 concurrent exercise sessions varying by aerobic exercise modality. Resistance training component remained constant. 4 exercises, 4 sets, performed to failure at 10RM, performed as 2 supersets.	Indirect calorimetry. Exercise EE = 5.05 kcal/L O_2
Freitas, M. C. et al. (2019) [33]	11 (11 male)	27.5 ± 5.5	Resistance trained	Half squat session performed twice. 4 sets, performed to momentary failure at 80% 1RM.	Indirect calorimetry. Aerobic metabolism estimated using the oxygen uptake interval.

					Lactic anaerobic contribution estimated using net blood La^- accumulation.<br>Anaerobic alactic estimated using the fast phase of excess postexercise oxygen uptake, corresponding to the product of bi-exponential fast component amplitude and tau.<br>Total energy expenditure was the sum of aerobic, anaerobic lactic, and anaerobic alactic metabolism, converted to kJ, assuming 20.9 kJ/L O_2
Garnacho-Castano, M. V. et al. (2019) [88]	21 (21 male)	21.4 ± 1.5	Resistance trained	Single half squat session. 21 sets, 15 repetitions at a load corresponding to La^- threshold 1.	Indirect calorimetry. Exercise EE determined using the calorie equivalent of O_2 based on RER.
Garnacho-Castano, M. V. et al. (2021) [89]	19 (19 male)	21.7 ± 1.7	Resistance trained	Single half squat session. 21 sets, 15 repetitions at a load corresponding to La^- threshold 1.	Indirect calorimetry. Exercise EE determined using the calorie equivalent of O_2 based on RER.
Greer, B. K. et al. (2021) [90]	7 (7 female)	23.0 ± 3.0	Resistance trained	Single resistance training session. 5 exercises, 4 sets, 30s per set at 80% 1RM.	Indirect calorimetry. Exercise EE calculated using non-protein respiratory quotients.
Greer, B. K. et al. (2015) [91]	10 (10 male)	22.0 ± 2.0	Recreationally trained	Single circuit resistance training session. 4 stations per round, repetitions performed at 60% 1RM until fatigue. Circuit repeated continuously for 45 minutes.	Indirect calorimetry. Exercise EE calculated = 3.941 x VO_2 + 1.106 x VCO_2
Gremeaux, V. et al. (2012) [92]	62 (22 female)	53.3 ± 9.7	Resistance trained	Circuit resistance training session. 15-20 repetitions for each muscle group at 50% 1RM, 20 minutes total duration.	Estimated using a standard expenditure per minute. Exercise EE = 19.97 kcal/minute of resistance exercise.
Guiraud, T. et al. (2015) [93]	20 (1 female)	53.1 ± 9.9	Clinical	2 whole body isometric strength training sessions on an oscillating platform, varying by intensity. 6 exercise blocks, each block was 8 contractions of 6 seconds, repeated twice. Sessions performed at 40% or 70% maximal voluntary contraction.	Indirect calorimetry. Exercise EE = (3.9 x VO_2 + 1.1 x VCO_2) x 1.44
Gutierrez, J. et al. (2015) [94]	7 (7 male)	23.0 ± 1.2	Untrained	Single circuit resistance training session. 8 stations per round, 10 repetitions per station at 7-8/10 on RPE scale, 40 minutes total duration.	Whole room indirect calorimetry. Exercise EE = 3.941 x VO_2 + 1.106 x VCO_2
Haddock, B. L. & Wilkin, L. D. (2006) [95]	15 (15 female)	24.2 ± 1.2	Resistance trained	2 circuit resistance training sessions, varying by rounds number. 1 or 3 rounds, 9 exercises per round, repetitions performed to fatigue at 8RM.	Indirect calorimetry. Exercise EE estimated using VO_2 and RER.
Hajj-Boutros, G. et al. (2022) [96]	60 (30 female)	24.9 ± 3.0	Recreationally trained	Single resistance training session. 3 exercises, 3 sets, 10 repetitions per exercise at a moderate-vigorous self-selected load.	Indirect calorimetry.<br>3 wearable devices (Apple Watch 6 Version 7.0, Polar Vantage V Firmware 5.1.8, Fitbit Sense Version 128.4.17)
Haltom, R. W. et al. (1999) [97]	7 (7 male)	26.9 ± 1.4	Resistance trained	2 circuit resistance training sessions, varying by rest interval (20 seconds or 60 seconds). 2 rounds, 8 stations per round, 20 repetitions per station at 75% 20RM.	Indirect calorimetry. Exercise EE = 5.05 kcal/L O_2
Harris, N. K. et al. (2017) [99]	17 (9 female)	Female: 12.9 ± 0.3<br>Male: 13.0 ± 0.3	Recreationally trained	Single circuit resistance training session. 4 rounds, 3 stations per round, 30 seconds per set using body weight exercises.	Indirect calorimetry. Exercise aerobic EE = 5.05 kcal/L O_2<br>Rest EE = 4.7 kcal/L O_2<br>Δ blood $[La^-]$. 3 mL O_2 x mass (kg) x $\Delta[La^-]$ (mmol)<br>Total EE = aerobic EE + rest EE + lactate EE
Harris, N. K. et al. (2016) [100]	10 (10 male)	23.6 ± 7.5	Resistance trained	2 resistance training sessions, varying by exercises. Traditional: 4 exercises (squat, deadlift, bench press, clean), 3 sets, 10 repetitions per set at 75% 1RM;	Indirect calorimetry. Exercise EE estimated through stoichiometry via carbohydrate and fat oxidation.

				Strongman: 4 exercises (sled drag, farmers walk, 1 arm dumbbell clean and press, tyre flip) 3 sets, repetition and load varied by exercise.	
Harris, N. et al. (2018) [98]	12 (12 females)	30.1 ± 5.8	Resistance trained	Group exercise class (body pump). 4 major movements performed, 60-minute duration.	Heart rate monitor. Exercise EE estimated using individual heart rate-VO_2 regression equation, 5.024 kcal/L O_2
Heden et al., (2011) [101]	8 (8 males)	21.0 ± 1.5	Untrained	2 sessions, varying by number of sets. 10 exercises, 10 reps, 1 or 3 sets, 10RM load. Exercises performed as mini circuits: 3 exercises, 3 exercises, 4 exercises.	Indirect calorimetry
Hu, J. et al. (2021) [102]	14 (7 female)	Female: 23.0 ± 3.2<br>Male: 22.0 ± 2.4	Recreationally trained	Single immersive virtual reality technology with cable resistance training system. 30 minutes exergame session. 6 exercises linked to in-game attacks. 12-14 repetitions completed per set at 60-70% 1RM.	Indirect calorimetry. Exercise EE = 3.941 x VO_2 + 1.106 x VCO_2
Hulsey, C. R. et al. (2012) [103]	13 (2 female)	21.4 ± 2.1	Recreationally trained	Single kettlebell swing routine. 10-minute routine, 35 seconds swing, 25 seconds rest. Men used 16kg, women used 8kg.	Indirect calorimetry
Hunter, G. R. et al. (2013)[#] [104]	72 (72 female)	60-74	Untrained	10 exercises, 2 sets, 10 repetitions per exercise at 80% 1RM.	Indirect calorimetry. Exercise EE = 5 kcal/L O_2
Hunter, G. R. et al. (2003) [105]	7 (7 male)	24.3 ± 3.8	Resistance trained	2 resistance training sessions varying by velocity of movement. Traditional training: 10 exercises, 2 sets, 8 repetitions per set at 65% 1RM, 1 second concentric contraction, 1 second eccentric contraction; Super slow training: 10 exercises, 1 set, 8 repetitions per set at 25% 1RM, 10 seconds concentric contraction, 5 seconds eccentric contraction.	Indirect calorimetry. Exercise EE = 5 kcal/L O_2<br>Δ blood $[La^-]$. 0.02698 kcal x mass (kg) x $\Delta[La^-]$ (mmol)<br>Total EE = aerobic EE + lactate EE
Jacobs, P. L. et al. (2002) [106]	6 (6 male)	35.7 ± 9.8	Clinical	Circuit resistance training session. Isoinertial resistance exercise interspersed with periods of high cadence low resistance arm ergometry. 3 rounds, 3 pairs of resistance exercises per round, 10 repetitions per exercise at 60% 1RM, 2 minutes of arm cranking between resistance exercise pairs.	Indirect calorimetry. Exercise EE = 5 kcal/L O_2
Jarvinen, L. et al. (2022) [107]	15 (5 females)	25.2 ± 3.3	Recreationally trained	2 circuit resistance exercise sessions, varying by intensity. 2 rounds, 10 stations per round, maximum repetitions in 30 seconds per station at either 40% or 60% 1RM.	Indirect calorimetry. Exercise EE = 5.05 kcal/L O_2<br>Resting EE = 4.64 kcal/L O_2<br>Δ blood $[La^-]$. 3 mL O_2 x mass (kg) x $\Delta[La^-]$ (mmol), anaerobic EE = 5.05 kcal/L O_2<br>Total EE = aerobic EE + rest EE + anaerobic EE
Joao, G. A. et al. (2021) [108]	15 (15 male)	22.9 ± 2.6	Resistance trained	3 resistance training sessions varying by intensity. Low: 8 exercises, 2 sets, 15 repetitions per set at 60% 1RM; Intermediate: 8 exercises, 3 sets, 10 repetitions per set at 75% 1RM; High: 8 exercises, 6 sets, 5 exercises per set at 90% 1RM.	Indirect calorimetry. Exercise EE = 5.05 kcal/L O_2<br>Δ blood $[La^-]$. 3 mL O_2 x mass (kg) x $\Delta[La^-]$ (mmol), anaerobic EE = 5.05 kcal/L O_2<br>Total EE = aerobic EE + anaerobic EE
Jun Liang, O. & Brownlee, I. A. (2017) [109]	9 (9 females)	23.0 ± 1.9	Athlete	Participants performed their usual resistance training program. Program not described.	Sensewear armband
Kalb, J. S. & Hunter, G. R. (1991) [110]	7 (5 female)	Female: 20.8<br>Male: 34.5	Resistance trained	4 single exercise sessions, varying by intensity. 4 sets, 10 repetitions per set at 60% 1RM; 4 sets, 5 repetitions per set at 80% 1RM. Back squat and overhead press.	Indirect calorimetry. Exercise EE = 5 kcal/L O_2

Katch, F. I. et al. (1985) [111]	20 (20 male)	23.7 ± 4.4	Untrained	Single resistance training session performed twice, using a hydraulic resistance exercise machine providing resistance during both directions of movement. 3 exercises, 3 sets, 20 seconds of maximum repetitions per set performed using maximal force.	Indirect calorimetry. Exercise EE = VO_2 x caloric equivalent per L O_2 at the given RER
Keim, N. L. et al. (1996)* [112]	15 (15 female), 7 performed RT	31.0 ± 3.0	Untrained	Low intensity resistance training session. 5-6 exercises, 1 set, 8 repetitions per set at 50-60% 1RM.	Indirect calorimetry. Exercise EE = 3.941 x VO_2 + 1.106 x VCO_2
Kelleher, A. R. et al. (2010) [113]	10 (10 male)	21.7 ± 2.1	Resistance trained	2 resistance training sessions, varying by set structure. Superset: 3 pairs of exercises performed as supersets, 4 sets, repetitions performed to volitional fatigue at 70% 1RM; Traditional: 6 exercises, 4 sets, repetitions performed to match Superset volume at 70% 1RM.	Indirect calorimetry. Exercise EE = [(4.21 x VCO_2 – 2.962 x VO_2) x 4.07 kcal] + [(1.695 x VO_2 – 1.701 x VCO_2) x 9.75 kcal]
Kemmler, W. et al. (2012) [114]	19 (19 male)	26.3 ± 4.5	Trained	Single resistance training session performed with and without electromyostimulation. 5 exercises, 2 sets, 8 repetitions per set, no additional resistance.	Indirect calorimetry
Kinnunen, H. et al. (2019) [115]	15 (15 male)	30.0 ± 6.0	Recreationally trained	Resistance training sessions assessed throughout a 12-week intervention. 6-8 exercises per session, 2-5 sets, 3-10 repetitions per set at 40-95% 1RM.	Wrist-worn motion sensor (Polar Active)<br>Heart rate monitor (Polar RS800CX)
Koerhuis, C. L. et al (2003) [116]	11 (2 female)	22.4 ± 1.6	Athlete	Single exercise resistance training session. Isometric calf raises, performed on alternate legs for 3 periods of 5 minutes, with contraction durations of 15, 10, and 5 seconds.	Indirect calorimetry. Exercise EE = (255.5 + 94.5 x RER) x VO_2
Lundstrom, E. A. et al. (2023) [25]	25 (15 female)	Female: 19.2 ± 0.9<br>Male: 20.1 ± 0.9	Athlete	Participants performed their usual resistance training program. Program not described.	Combined heart rate accelerometer (WHOOP)
Lyristakis, P. et al. (2019) [117]	10 (10 male)	29.4 ± 10.2	Resistance trained	Resistance training session performed on 3 occasions. 2 exercises, 3 sets, 10 repetitions per set at 60% 1RM.	Heart rate monitor (Suunto T6D)<br>Indirect calorimetry. aerobic exercise EE = 21.1 kJ/L O_2, rest EE = 19.6 kJ/L O_2<br>Δ blood $[La^-]$. 3 mL O_2 x mass (kg) x $\Delta[La^-]$ (mmol), anaerobic EE = 21.1 kJ/L O_2
Lytle, J. R. et al. (2019) [36]	52 (25 female)	Female: 36.0 ± 13.2<br>Male: 30.0 ± 11.3	Recreationally trained	Single resistance training session. 7 exercises, 2-3 sets, 8-12 repetitions at 70% 1RM.	Indirect calorimetry. Exercise EE estimated using VO_2 and RER
Magkos, F. et al. (2008) [118]	7 (7 male)	25.0 ± 1.0	Untrained	Single isokinetic dynamometer resistance training session. 12 exercises, 3 sets, 10 repetitions per set at 80% peak torque. Each exercise performed separately for each limb.	Indirect calorimetry.
Magosso, R. F. et al. (2013) [119]	10 (10 male)	20.3 ± 4.2	Resistance trained	Two single exercise resistance training sessions. 4 sets, 10 repetitions at 70% 1RM on bench press or leg press.	Indirect calorimetry. Exercise EE = 21.1 kJ/L O_2<br>Δ blood $[La^-]$. 3 mL O_2 x mass (kg) x $\Delta[La^-]$ (mmol), anaerobic EE = 21.1 kJ/L O_2<br>Total EE = aerobic EE + anaerobic EE
Marzolini, S. et al. (2012) [120]	16 (6 female)	60.8 ± 13.7	Clinical	Single combined aerobic (30 minutes) and resistance training session (30 minutes) performed during stroke rehabilitation program. 1-2 sets per exercise, 10-15 repetitions per set at 50-60% 1RM.	Indirect calorimetry. Exercise EE = 5.05 kcal/L O_2

Mazzetti, S. A. et al. (2011)a [121]	10 (10 male)	22.0 ± 3.6	Resistance trained	4 resistance training sessions varying by intensity.<br>Light-48: 2 exercises, 4 sets, 10 repetitions per set at 48% 1RM.<br>Moderate-60: 2 exercises, 4 sets, 8 repetitions per set at 60% 1RM.<br>Heavy-72: 2 exercises, 5 sets, 5 exercises per set at 72% 1RM.<br>Heavy-72 matched: 2 exercises, 4 sets, 6.5 repetitions per set at 72% 1RM	Indirect calorimetry. Exercise EE = 4.9 kcal/L O_2<br>Δ blood [La^-]. Anaerobic EE = Δ [La^-] (mmol) x mass (kg) x 0.02698 kcal<br>Total EE = aerobic EE + anaerobic EE
Mazzetti, S. et al. (2007) [37]	9 (9 male)	20.2 ± 2.5	Resistance trained	3 single exercise resistance training sessions varying by contraction velocity.<br>Slow: 4 sets, 8 repetitions per set at 60% 1RM, 2 seconds eccentric, 2 seconds concentric.<br>Explosive: 4 sets, 8 repetitions per set at 60% 1RM, 2 seconds eccentric, maximal velocity concentric.<br>Heavy explosive: 6 sets, 4 repetitions at 80% 1RM, 2 seconds eccentric, maximal velocity concentric.	Indirect calorimetry. Exercise EE = 3.941 x VO_2 + 1.106 x VCO_2<br>Δ blood [La^-]. Anaerobic EE = Δ [La^-] (mmol) x mass (kg) x 0.02698 kcal<br>Total EE = aerobic EE + anaerobic EE
Mazzetti, S. et al. (2011)b [122]	14 (14 male; 7 trained, 7 untrained)	Trained: 21.9 ± 2.1<br>Untrained: 20.1 ± 2.2	Resistance trained and untrained	3 resistance training sessions varying by contraction velocity.<br>Slow: 7 exercises, 3 sets, 2-8 repetitions per set, 2 seconds eccentric, 2 seconds concentric.<br>Recreational: 7 exercises, 2-8 repetitions per set, 1 second eccentric, 1 second concentric.<br>Maximal: 7 exercises, 2-8 repetitions per set, 2 seconds eccentric, maximal velocity concentric.	Indirect calorimetry. Exercise EE = 5 kcal/L O_2<br>Δ blood [La^-]. Anaerobic EE = Δ [La^-] (mmol) x mass (kg) x 0.02698 kcal<br>Total EE = aerobic EE + anaerobic EE
McGuire, A. et al. (2023) [194]	30 (30 male)	24.1 ± 3.8	Athlete	Participants performed their usual resistance training program. Program not described.	Training log with assigned MET values from compendium of activities.
McMillan, D. W. et al. (2021) [123]	10 (10 male)	39.0 ± 10.0	Clinical	Single resistance training circuit. 3 rounds, 6 stations per round, 10 repetitions per station at 60% 1RM. Low resistance arm cranking performed after every second station.	Indirect calorimetry. Stoichiometric equations used to calculate EE.
Melanson, E. L. et al. (2005) [124]	7 (7 female)	40.0 ± 8.0	Recreationally trained	Single resistance training session. 10 exercises, 4 sets, 10 repetitions per set at 70% 1RM, final set performed to failure.	Whole room indirect calorimeter. Aerobic EE estimated using VO_2 and RER
Melanson, E. L. et al. (2002) [125]	10 (10 male)	31.0 ± 7.0	Recreationally trained	Single resistance training session. 10 exercises, 4 sets, 10 repetitions per set at 70% 1RM, final set performed to failure.	Whole room indirect calorimeter. Aerobic EE estimated using VO_2 and RER
Melby, C. et al. (1993) [126]	2 (2 male)	Not reported	Resistance trained	Single resistance training session. 10 exercises performed as supersets, 5 sets, 8-10 repetitions per exercise performed to failure at 70% 1RM.	Indirect calorimetry. Exercise EE = 5 kcal/L O_2
Michalski, A. C. et al. (2023) [195]	7 (3 female)	58.0 ± 3.0	Clinical	Circuit resistance training session performed twice. 3 rounds, 10 exercises, 15 repetitions at 100% 15RM.	Indirect calorimetry. Exercise EE = 3.941 x VO_2 + 1.106 x VCO_2
Monteiro, A. G. et al. (2008) [127]	25 (15 female)	Female: 24.2 ± 6.6<br>Male: 26.4 ± 4.7	Resistance trained	2 circuit resistance training sessions varying by inclusion of aerobic exercise.<br>Circuit weight training: 1 round, 8 stations per round, 60 seconds per station, 2-6kg per exercise.	Indirect calorimetry. Exercise EE = 5 kcal/L O_2

				Combined circuit training: 1 round, 8 stations per round, 30 seconds resistance exercise + 30 seconds of treadmill running per station, 2-6kg per exercise.	
Mookerjee, S. et al. (2016) [128]	24 (12 female)	Female: 21.0 ± 0.7<br>Male: 21.8 ± 1.6	Resistance trained	2 resistance training session varying by number of sets.<br>Single set: 5 exercises, 1 set, 10 repetitions per set at 70% 1RM.<br>Multiple sets: 5 exercises, 3 sets, 10 repetitions per set at 70% 1RM.	Indirect calorimetry. Exercise EE = 3.781 x VO_2 + 1.237 x VCO_2
Moon, J. M. et al. (2021) [129]	33 (33 female: 13 basketball, 20 la crosse)	Basketball: 19.8 ± 1.3<br>La crosse: 20.4 ± 1.8	Athlete	Participants performed their usual resistance training program. Program not described.	Combined heart rate and accelerometer (Actiheart).
Morgan, B. et al. (2003) [130]	15 (7 female)	Female: 20.7 ± 1.0<br>Male: 22.8 ± 2.9	Resistance trained	2 resistance training sessions varying by intensity and duration.<br>Heavy: 8 exercises, 2 sets, 8 repetitions per set at 100% 8RM.<br>Light: 8 exercises, 2 sets, 15 repetitions per set at 85% 8RM.	Indirect calorimetry. Exercise EE = (3.781 x VO_2 + 1.237 x VCO_2) x 4.2
Morris, C. E. et al. (2019) [131]	47 (25 females)	Female: 26.9 ± 11.5<br>Male: 29.5 ± 11.9	Recreationally trained	Single circuit resistance exercise session. 15 minutes, 7 stations, 12 repetitions per station, as many rounds as possible.	Indirect calorimetry.<br>5 different wearable devices (Fitbit Charge Heart Rate, Nike Fuel Band, Fitbit One, Jawbone UP Move, Actigraph GTX3)
Moss, S. L. et al. (2021) [132]	13 (13 female)	23.7 ± 3.4	Athlete	Participants performed their usual resistance training program. Program not described.	Training log with assigned MET values from compendium of activities.
Mukaimoto, T. & Ohno, M. (2012) [133]	11 (11 male)	Not reported	Resistance trained	3 circuit resistance training sessions varying by intensity and repetition velocity.<br>Light-slow: 3 rounds, 4 stations, each round performed to fatigue at 50% 1RM, 4 seconds eccentric, 4 seconds concentric.<br>Heavy-normal: 3 rounds, 4 stations, each round performed to fatigue at 50% 1RM, 1 second eccentric, 1 second concentric.<br>Light-normal: 3 rounds, 4 stations, each round performed to fatigue at 50% 1RM, 1 second eccentric, 1 second concentric.	Indirect calorimetry. Exercise EE = VO_2 x (15.480 + 5.550 x RER) / 4.184 / 1000
Munch, G. W. et al. (2018)* [134]	28 (4 female)	59.0 ± 3.0	Clinical	Single resistance trained session performed twice. 4 exercises, 4 sets, 30 seconds per set (15-20 repetitions) at 40% 1RM.	Indirect calorimetry
Nakagata, T. et al. (2018) [135]	8 (8 male)	23.4 ± 1.8	Resistance trained	Single body weight resistance training session. 6 exercises, 3 sets, 10 repetitions per set.	Indirect calorimetry. Exercise EE = 3.941 x VO_2 + 1.106 x VCO_2
Nakagata, T. et al. (2019) [136]	20 older participants (7 female); 15 young participants (15 male)	Older women: 69.3 ± 3.3<br>Older men: 71.6 ± 5.4<br>Younger men: 21.7 ± 1.7	Resistance trained	Single body weight resistance training session. 4 exercises, 3 sets, 10 repetitions per set.	Indirect calorimetry. Exercise EE = 5 kcal/L O_2<br>Rest EE = 4.7 kcal/L O_2

Nakagata, T. et al. (2022) [137]	15 (15 male)	23.2 ± 2.0	Resistance trained	Slow movement body weight resistance exercise performed as a multistage test. 3 exercises performed (heel raise, squat, push up). Each stage lasted 5 minutes and had the exercise performed at a different frequency: 1, 2, 3, 4, 5, and 6 repetitions per minute.	Indirect calorimetry. Exercise EE = 5 kcal/L O_2
Nunez, T. P. et al. (2020) [138]	14 (14 male)	25.7 ± 4.4	Resistance trained	2 circuit resistance training sessions combined with HIIT. Sessions performed as either HIIT then circuit, or HIIT integrated into circuit. 3 rounds, 9 stations, 30 seconds per station.	Indirect calorimetry
Ortego, A. R. et al. (2009)* [139]	20 (10 female)	Female: 23.8 ± 5.5<br>Male: 23.4 ± 1.6	Resistance trained	Single circuit resistance training session. 3 rounds, 6 stations, 10 repetitions per station at 12RM.	Indirect calorimetry.
Paduan Joaquim, D. et al. (2018) [140]	17 (8 female)	Visually impaired: 26.0 ± 6.2<br>Amputee: 27.0 ± 8.5<br>Cerebral palsy: 24.0 ± 7.5	Athlete	Participants performed their usual resistance training program. Program not described.	Wearable accelerometer (Actical)
Pafili, Z. K. et al. (2010) [141]	9 (9 male)	27.2 ± 1.1	Untrained	Single exercise resistance training session. 8 sets, 6 repetitions at 100% 6RM.	Indirect calorimetry. Exercise EE = 21.1 kJ/L O_2<br>Rest EE = 19.6 kJ/L O_2<br>Δ blood $[La^-]$. 3 mL O_2 x mass (kg) x $\Delta[La^-]$ (mmol), anaerobic EE = 21.1 kJ/L O_2<br>Total EE = aerobic EE + anaerobic EE
Parmar, P. et al. (2015) [142]	36 (19 female)	Not reported	Not described	3 different single exercise sessions varying by contraction type.<br>CO2: concentric contraction only, 2 sets, 8 repetitions per set.<br>CE2: concentric and eccentric contraction, 2 sets, 8 repetitions per set.<br>CO4: concentric contraction only, 4 sets, 8 repetitions per set.<br>CE4: concentric and eccentric contractions, 4 sets, 8 repetitions per set.	Indirect calorimetry. Exercise EE = 5 kcal/L O_2
Petitt, D. S. et al. (2003) [143]	14 (4 female)	24.3 ± 2.9	Resistance trained	Single resistance training session. 10 exercises, 3 sets, 10 repetitions per set at 100% 10RM.	Indirect calorimetry
Phillips, W. T. & Ziuraitis, J. R. (2003) [144]	12 (6 female)	Female: 27.0 ± 4.0<br>Male: 25.2 ± 3.7	Resistance trained	Single resistance training session. 8 exercises, 1 set, 15 repetitions per set at 100% 15RM.	Indirect calorimetry. Exercise EE = 5.05 kcal/L O_2
Phillips, W. T. & Ziuraitis, J. R. (2004) [145]	10 (5 females)	Female: 73.8 ± 6.4<br>Male: 72.4 ± 4.9	Resistance trained	Single resistance training session. 8 exercises, 1 set, 15 repetitions per set at 100% 15RM.	Indirect calorimetry. Exercise EE = 5.05 kcal/L O_2
Quintero, A. P. et al. (2018) [146]	36 (36 male)	Control: 24.7 ± 3.4<br>Resistance training: 22.8 ± 3.1<br>Interval training: 24.5 ± 3.7	Untrained	2 resistance training sessions with or without HIIT.<br>Resistance training only: 6 exercises, 12-15 repetitions per set at 50-70% 1RM. Session continued until 400-500 kcal expended.<br>Resistance training with HIIT: 6 exercises, 12-15 repetitions per set at 50-70% 1RM, with 2 x 4-minute	Indirect calorimetry

		Resistance + interval training: 22.2 ± 3.4		bouts of 65% HRmax. Session continued until 400-500 kcal expended	
Rangan, V. V. et al. (2011) [147]	All: 82; RT 28 (20 female); RT+AT: 26 (16 female)	Resistance training: 50.6 ± 12.4<br>Resistance + aerobic training: 47.5 ± 11.0	Untrained	Single resistance training session performed on 3 occasions. 8 exercises, 3 sets, maximum load lifted for 8-12 repetitions per set.	Wearable accelerometer (RT3)<br>Indirect calorimetry (n = 5)
Ratamess, N. A. et al. (2016) [148]	12 (12 male)	22.3 ± 1.6	Resistance trained	Single exercise (back squat) resistance training session performed on 4 occasions. 6 sets, 10 repetitions per set at 80% 1RM.	Indirect calorimetry. Exercise EE = 4.8-5.05 kcal/L O_2 dependent on RER.
Ratamess, N. A. et al. (2007) [149]	8 (8 male)	21.4 ± 2.4	Resistance trained	10 single exercise (bench press) resistance training sessions varying by load, repetitions, and rest interval. 5 sets, 10 repetitions per set at 75% 1RM or 5 repetitions per set at 85% 1RM, with rest intervals of 30 seconds, 1, 2, 3, or 5 minutes.	Indirect calorimetry. Exercise EE = 5 kcal/L O_2
Ratamess, N. A. et al. (2018) [150]	8 (8 male)	21.1 ± 1.0	Resistance trained	Single resistance training circuit. 3 rounds, 8 stations per round, 20 seconds per station (as many repetitions as possible) with load of 11kg, 20kg, or 48kg sandbag dependent on exercise.	Indirect calorimetry. Exercise EE = 4.86-5.05 kcal/L O_2 dependent on RER.<br>Δ blood [La^-]. 3 mL O_2 x mass (kg) x Δ[La^-] (mmol), anaerobic EE = 21.1 kJ/L O_2<br>Total EE = aerobic EE + anaerobic EE
Ratamess, N. A. et al. (2014) [151]	17 (17 male)	21.6 ± 2.3	Resistance trained	3 resistance training sessions varying by rest interval. 2 exercises, 5 sets, up to 10 repetitions per set at 75% 1RM. 1-, 2-, or 3-minutes rest between sets.	Indirect calorimetry. Exercise EE = 5.05 kcal/L O_2 based on RER
Ratamess, N. A. et al. (2015) [152]	10 (10 male)	20.6 ± 1.3	Resistance trained	13 single exercise sessions. 3 sets, 10-20 repetitions or 30 seconds per set at 75% 1RM or body weight or battle rope.	Indirect calorimetry. Exercise EE = 4.86-5.05 kcal/L O_2 dependent on RER.
Rawson, E. S. & Walsh, T. M. (2010) [153]	30 (15 female)	Female: 21.2 ± 1.4<br>Male: 22.0 ± 2.3	Resistance trained	Single resistance training session. 8 exercises, 2 sets, 8-10 repetitions per set at 100% 10RM.	Indirect calorimetry
Realzola, R. A. et al. (2021) [154]	18 (9 female)	Female: 22.8 ± 3.9<br>Male: 24.1 ± 3.7	Resistance trained	2 resistance training sessions varying by set structure. Reciprocal superset: 3 clusters of 2 exercises performed as supersets, 4 sets, 12-15 repetitions per set performed to failure at 75% 10RM.<br>Traditional: 6 exercises, 4 sets, 12-15 repetitions per set performed to failure at 75% 10RM.	Indirect calorimetry. Exercise EE = 20.9 kJ/L O_2<br>Δ blood [La^-]. 3 mL O_2 x mass (kg) x Δ[La^-] (mmol), anaerobic EE = 21.1 kJ/L O_2<br>Total EE = aerobic EE + anaerobic EE
Reed, J. L. et al (2013) [155]	19 (19 female)	19.2 ± 0.3	Athlete	Participants performed their usual resistance training program. Program not described.	Heart rate monitor
Reed, J. L. et al (2014) [156]	19 (19 female). LEA n=5; NEA n=14	LEA: 19.0 ± 1.0<br>NEA: 19.0 ± 1.0	Athlete	Participants performed their usual resistance training program. Program not described.	Heart rate monitor
Reeve, M. D. et al. (2014) [157]	18 (7 female)	Female: 22.1 ± 4.2<br>Male: 23.1 ± 5.0	Resistance trained	Single resistance training session performed on 2 occasions. 9 exercises, 3 sets, 10 repetitions per set at 70% 1RM.	Indirect calorimetry<br>2 wearable sensors (Sensewear armband, BodyMedia FIT)
Reis, V. M. et al. (2017) [38]	58 (58 male)	27.5 ± 4.9	Resistance trained	8 single exercise resistance training sessions. Each exercise performed 5 times at varied intensities. 1 set,	Indirect calorimetry. Exercise EE = 5 kcal/L O_2

				4 minutes continuous (60 repetitions) at 12%, 16%, 20%, 24% 1RM. 1 set performed to exhaustion at 80% 1RM.	Anaerobic EE calculated by accumulated O_2 deficit method
Reis, V. M. et al. (2019) [158]	56 (56 male)	27.5 ± 4.9	Resistance trained	8 single exercise resistance training sessions. Each exercise performed 4 times at varied intensities. 1 set, 4 minutes continuous (60 repetitions) at 12%, 16%, 20%, 24% 1RM.	Indirect calorimetry. Exercise EE = 5 kcal/L O_2<br>Heart rate monitor
Robergs, R. A. et al. (2007) [159]	30 (30 male). 23 bench press, 20 parallel squat	Bench press: 23.6 ± 4.6<br>Parallel squat: 23.3 ± 6.9	Resistance trained	2 single exercise resistance training sessions. 5 bouts performed for 5 minutes continuously (100 repetitions) at 31-57% 1RM.	Indirect calorimetry. Exercise EE = 5.05 kcal/L O_2
Roberson, K. B. et al. (2017) [160]	15 (8 female)	Female: 20.0 ± 0.9<br>Male: 21.1 ± 0.5	Resistance trained	3 circuit resistance training sessions varying by intensity and velocity.<br>High load controlled: 3 rounds, 7 stations, 6 repetitions per station at 80% 1RM, 2 seconds eccentric, 2 seconds concentric.<br>High load explosive: 3 rounds, 7 stations, 6 repetitions per station at 80% 1RM, 2 seconds eccentric, maximal velocity concentric.<br>Moderate load explosive: 3 rounds, 7 stations, 12 repetitions per station at 50% 1RM, 2 seconds eccentric, maximal velocity concentric.	Indirect calorimetry. Exercise EE = 3.941 x VO_2 + 1.106 x VCO_2<br>Δ blood [La^-]. 3 mL O_2 x mass (kg) x Δ[La^-] (mmol) / 1000. Anaerobic EE = 5 kcal/L O_2<br>Total EE = aerobic EE + anaerobic EE
Rustaden, A. M. et al. (2020) [161]	18 (18 female)	35.4 ± 10.2	Untrained	Body pump group class: 1 hour, 50-100 repetitions per muscle group, 1kg, 2.5kg, or 5kg weights on a 1.25kg bar.<br>Heavy resistance training session: 12 exercises, 2-4 sets, 8 repetitions per set at 100% 8RM.	Indirect calorimetry. Exercise EE = 5 kcal/L O_2
Salagaras, B. S. et al. (2021)a [162]	14 (14 male)	24.0 ± 4.0	Athlete	Participants performed their usual resistance training program. Program not described.	Wearable triaxial accelerometer (GeneActive)
Salagaras, B. S. et al. (2021)b [163]	19 (19 males)	24.0 ± 4.0	Athlete	Participants performed their usual resistance training program. Program not described.	Wearable triaxial accelerometer (GeneActive)
Santa-Clara, H. et al. (2018) [164]	12 (12 male)	62.6 ± 8.5	Clinical	Cardiac rehabilitation consisting of aerobic and recreational activity with resistance training. 8 exercises, 2 sets, 8-12 repetitions per set at 50-60% 1RM, plus 2 sets of abdominal exercise and 2 sets of lower back exercise.	Indirect calorimetry. Exercise EE = 3.941 x VO_2 + 1.106 x VCO_2
Santos, D. A. et al. (2014) [165]	12 (8 female)	Female: 16.5 ± 0.5<br>Male: 16.5 ± 0.6	Athlete	Participants performed their usual resistance training program. Program not described.	Activity EE calculated as total EE (doubly labelled water) - resting EE (indirect calorimetry)<br>Combined heart rate and accelerometer (Actiheart)
Sarafian, D. et al. (2016) [166]	26 (13 female)	23.0 ± 1.0	Not described	8 cycles of intermittent isometric leg press contractions varying by load. 30 seconds contraction, 30 seconds rest, 8 contractions at 5 different loads.	Indirect calorimetry. Exercise EE = 5.68 x VO_2 + 1.59 x VCO_2 – 2.17 x N_u<br>Where N_u is total nitrogen excreted, assumed to be 13 g/24 hour
Schaal, K. et al. (2021) [167]	16 (16 female; 9 well adapted, 7 non-	Well adapted: 29.4 ± 1.6<br>Non-functional overreaching: 27.7 ± 2.3	Trained	Participants performed their usual resistance training program. Program not described.	Combined heart rate and accelerometer (Actiheart)

	functional overreach)				
Scheffer, J. H. et al. (2023) [196]	25 (25 female)	24.8 ± 2.7	Athlete	Participants performed their usual resistance training program. Program not described.	Wrist-worn GPS watch paired with heart rate monitor
Scott, C. B. (2006) [168]	11 (6 female)	Female: 24.6 ± 7.2<br>Male: 25.8 ± 8.5	Resistance trained	2 resistance training sessions varying by intensity. 3 exercises, 2 sets, 6-10 repetitions per set at 80% 1RM or repetitions to failure at 60% 1RM.	Indirect calorimetry. Exercise EE = 21.1 kJ/L O_2<br>Δ blood [La^-]. 3 mL O_2 x mass (kg) x Δ[La^-] (mmol), anaerobic EE = 21.1 kJ/L O_2<br>Total EE = aerobic EE + anaerobic EE
Scott, C. B. (2012) [31]	10 (10 male)	23.2 ± 3.1	Resistance trained	3 single exercise resistance training sessions varying by velocity. 3 sets, 5 repetitions per set at 70% 1RM, 1.5 seconds eccentric, 1.5 seconds concentric, or 4 seconds eccentric, 1 second concentric, or 1 second eccentric, 4 seconds concentric.	Indirect calorimetry. Exercise EE = 21.1 kJ/L O_2<br>Δ blood [La^-]. 3 mL O_2 x mass (kg) x Δ[La^-] (mmol), anaerobic EE = 21.1 kJ/L O_2<br>Total EE = aerobic EE + anaerobic EE
Scott, C. B. et al. (2009) [30]	8 (4 female)	28.4 ± 9.0	Resistance trained	3 single exercise (bench press) resistance training sessions varying by repetition number. 3 sets, 7, 14, or 21 repetitions at 50% 1RM.	Indirect calorimetry. Exercise EE = 21.1 kJ/L O_2<br>Δ blood [La^-]. 3 mL O_2 x mass (kg) x Δ[La^-] (mmol), anaerobic EE = 21.1 kJ/L O_2<br>Total EE = aerobic EE + anaerobic EE
Scott, C. B. et al. (2011)a [27]	10 (10 male)	24.3 ± 3.3	Resistance trained	3 single exercise (bench press) resistance training sessions varying by intensity. 2 sets performed to fatigue at 70%, 80%, 90% 1RM.	Indirect calorimetry. Exercise EE = 21.1 kJ/L O_2<br>Δ blood [La^-]. 3 mL O_2 x mass (kg) x Δ[La^-] (mmol), anaerobic EE = 21.1 kJ/L O_2<br>Total EE = aerobic EE + anaerobic EE
Scott, C. B. et al. (2011)b [28]	13 (13 male)	23.8 ± 2.1	Resistance trained	6 single exercise (bench press) resistance training sessions varying by load. 1 set performed to fatigue at 36.4kg, 45.5kg, 54.5kg (endurance loads), and 70%, 80%, 90% 1RM (strength loads)	Indirect calorimetry. Exercise EE = 21.1 kJ/L O_2<br>Δ blood [La^-]. 3 mL O_2 x mass (kg) x Δ[La^-] (mmol), anaerobic EE = 21.1 kJ/L O_2<br>Total EE = aerobic EE + anaerobic EE
Scott, J. M. et al. (2014) [169]	9 (1 female)	34.5 ± 8.2	Not described	Single resistance training session measured during a 14-day bed rest trial. 4 exercises, 3 sets, 12 repetitions per set at 10RM, final set performed to fatigue.	Indirect calorimetry. Exercise EE calculated from VO_2 and RER
Seliger, V. et al. (1980) [166]	14 (14 male)	17.3 ± 0.8	Untrained	Single exercise (squat), single repetition resistance training. Single eccentric, concentric, and isometric contraction, each held for 7 seconds, at maximal resistance.	Indirect calorimetry
Seliger, V. et al. (1968) [171]	15 (15 male)	26.0 ± 4.6	Athlete	Single exercise (squat), single repetition resistance training performed on 2 occasions. Single eccentric contraction at 150% 1RM, single concentric contraction at 100% 1RM.	Indirect calorimetry
Shannon, K. A. et al. (2005) [172]	10 (6 female)	Female: 24.1 ± 1.2<br>Male: 24.5 ±0 .9	Resistance trained	3 sessions varying by number of sets (1 set, 3 sets, or 5 sets). 8 exercises, 10 repetitions per set at 70% 1RM.	Estimated using a standard rate of expenditure per minute (6.653 kcal/min resistance exercise)
Smith, L. E. et al. (2022) [197]	13 (8 female)	47.0 ± 10.0	Sedentary	Functional training session. 4 sets of 6-minute duration. 4 exercises per set, as many repetitions as possible.	Indirect calorimetry<br>Aerobic EE = 5.0 kcal/L O_2<br>Δ blood [La^-]. 3 mL O_2 x mass (kg) x Δ[La^-] (mmol), anaerobic EE = 5.0 kcal/L O_2<br>Total EE = aerobic EE + anaerobic EE
Stec, M. J. & Rawson, E. S. (2012) [173]	30 (15 female)	Female: 21.4 ± 1.2<br>Male: 22.1 ± 0.7	Resistance trained	Single session, 8 exercises, 2 sets, 8-10 repetitions at 10RM	Indirect calorimetry<br>Δ blood [La^-]. 3 mL O_2 x mass (kg) x Δ[La^-] (mmol), anaerobic EE = 5.0 kcal/L O_2<br>Total EE = aerobic EE + anaerobic EE

					4 wearable triaxial accelerometers (Actigraph GTX3, ActiTrainer)
Steele, J. et al. (2018) [174]	9 (9 male)	26.0 ± 10.0	Recreationally trained	Single exercise (leg press) resistance training. 4 sets, 12 repetitions per set at 12RM.	Indirect calorimetry.<br>Δ blood [La^-].
Taylor, H. L. et al. (2022) [14]	10 (10 male)	22.0 ± 8.0	Athlete	Participants performed their usual resistance training program. Program not described.	Training log with assigned MET values from compendium of activities.
Thornton, M. K. & Potteiger, J. A. (2002) [175]	14 (14 female)	26.8 ± 5.0	Resistance trained	2 resistance training sessions varying by intensity.<br>High intensity: 9 exercises, 2 sets, 8 repetitions per set at 85% 8RM.<br>Low intensity: 9 exercises, 2 sets, 15 repetitions per set at 45% 8RM.	Indirect calorimetry. Exercise EE = 5 kcal/L O_2
Thornton, M. K. et al. (2011) [176]	10 (10 female)	22.8 ± 2.7	Untrained	2 resistance training sessions varying by intensity.<br>High intensity: 9 exercises, 3 sets, 8 repetitions per set at 85% 8RM.<br>Low intensity: 9 exercises, 3 sets, 15 repetitions per set at 45% 8RM.	Indirect calorimetry. Exercise EE = 5 kcal/L O_2
Tornberg, A. B.; et al. (2017) [177]	30 (30 female)	EUM: 27.6 ± 5.6<br>SFHA: 26.1 ± 5.6	Athlete	Participants performed their usual resistance training program. Program not described.	Heart rate monitor and training log
Traversa, C. et al. (2022) [198]	15 (15 female)	20.5 ± 0.4	Athlete	Participants performed their usual resistance training program. Program not described.	Exercise EE calculated as (BMR x 0.05) x minutes of exercise
Trexler, E. T. et al. (2020) [178]	27 (27 male)	22.0 ± 4.0	Recreationally trained	Single exercise (leg extension) resistance training performed on 3 occasions. 1 set, 3 minutes of continuous concentric contractions at 25% MVC.	Indirect calorimetry
Trexler, E. T. et al. (2019) [179]	27 (27 male)	22.0 ± 4.0	Recreationally trained	Single exercise (leg extension) resistance training performed on 3 occasions. 5 sets, 30 maximal concentric repetitions per set.	Indirect calorimetry. Exercise EE = 3.9 x VO_2 + 1.1 x VCO_2 + 1440 minutes
van Etten, L. M et al. (1997) [180]	26 (26 male)	33.0 ± 6.0	Untrained	Resistance training session performed on 2 occasions. 5 exercises, 3 sets, 15 repetitions per set. Sequence performed twice.	Indirect calorimetry. Exercise EE = 3.941 x VO_2 + 1.106 x VCO_2
van Etten, L. M. et al. (1996) [181]	10 (10 male)	30.9 ± 5.1	Resistance trained	Resistance training session performed on 4 occasions. 5 exercises, 3 sets, 15 repetitions per set. Sequence performed twice. Loads increased between second and third sessions.	Indirect calorimetry. Exercise EE = 3.941 x VO_2 + 1.106 x VCO_2
Vandenbrink, D. N. et al. (2018) [182]	10 (5 female)	23.5 ± 3.7	Recreationally trained	Functional circuit resistance training. 2 rounds, 5 stations, 60 seconds per station, as many repetitions as possible, 6kg (females) or 10kg (males) load.	Indirect calorimetry
Vezina, J. W. et al. (2014) [183]	12 (12 male)	23.6 ± 2.9	Resistance trained	Circuit resistance training. 3 rounds, 4 stations per round, 60 seconds per station.	Indirect calorimetry. Exercise EE = 5 kcal/L O_2<br>Recovery EE = 4.7 kcal/L O_2
Vincent, H. K. et al. (2014) [184]	20 (6 female)	26.8 ± 5.9	Resistance trained	2 resistance training sessions varying by repetition and set structure.<br>Concentric focus: 6 exercises, 2 sets, 12 repetitions per set at 60% 1RM.<br>Eccentric focus: 6 exercises, 2 sets, 10 repetitions per set, eccentric contraction at 100% 1RM, concentric contraction at 50% 1RM.	Indirect calorimetry
Walker, E. J. et al. (2016) [185]	18 (18 male)	22.0 ± 3.0	Athlete	Participants performed their usual resistance training program. Program not described.	Sensewear armband

Wickham, J. B. et al. (2017) [186]	10 (5 female)	29.3 ± 8.0	Recreationally trained	Body pump exercise class.	Combined heart rate and accelerometer (Actiheart)
Williams, B. M. & Kraemer, R. R. (2015) [187]	8 (8 male)	21.5 ± 0.9	Trained	Kettlebell circuit training. 3 rounds, 4 stations per round, 20 seconds per station, 2 sets per station at 10-22kg load.	Indirect calorimetry
Wilmore, J. H. et al. (1978) [188]	40 (20 female)	Female: 20.3 ± 2.8<br>Male: 23.7 ± 4.4	Trained	Circuit resistance training performed on 2 occasions. 3 rounds, 10 stations per round, 20 seconds per station (15-18 repetitions) at 40% 1RM.	Indirect calorimetry. Exercise EE = 5.05 kcal/L O_2
Yoshida, A. et al. (2019) [189]	8 (8 female)	20.0 ± 1.0	Athlete	Participants performed their usual resistance training program. Program not described.	Training log with RPE documented. Exercise EE estimated by an RPE-EE curve created during a graded treadmill test
Zanders, B. R. et al. (2021) [190]	13 (13 female)	19.8 ± 1.3	Athlete	Participants performed their usual resistance training program. Program not described.	Combined heart rate and accelerometer (Actiheart)
Zotou, E. et al. (2010) [191]	5 (5 female)	32.0 ± 5.0	Untrained	Resistance training session. 12 exercises, 4 sets, 12 repetitions per set.	Indirect calorimetry

Equations used to calculate energy expenditure are provided when presented in studies. *, indicates age presented as mean ± SE; $^{\#}$, indicates age presented as range; EE, energy expenditure; La^-, lactate; RM, repetition maximum; EUM, eumenorrheic; RER, respiratory exchange ratio; SFHA, secondary functional hypothalamic amenorrhea; HIIT high intensity interval training; HRmax, heart rate maximum; MVC, maximal voluntary contraction; RPE, rating of perceived exertion

Supplementary Table 2. Quality appraisal assessment

Author	A clear description of the inclusion and exclusion criteria was provided	The trials were randomized	Treatment order was counterbalanced	The method used to generate the random allocation sequence was described	Sample size was justified	Attempts were made to control and/or monitor pre-trial conditions	Design incorporated measures of important baseline variables	Subjects were blinded	Investigators were blinded	Methods and successfulness of blinding were described	Details were provided regarding the inability of a subject to complete study requirements	Statistical methods described	Primary outcome measurement and variability reported	Results of statistical comparisons reported	Methods used to assess adverse effects described	Reproducibility of the primary outcome measure(s) was reported	A familiarization of the performance test was conducted	**Total score (%)**
Abboud, G. J. et al (2013)	1	1	0	0	1	1	1	NA	NA	NA	NA	1	1	1	NA	0	1	75.0
Aerenhouts, D. et al (2011)	0	NA	NA	NA	0	NA	NA	NA	NA	NA	NA	1	1	1	NA	NA	NA	60.0
Aniceto, R. R. et al (2013)	1	1	0	1	1	1	1	NA	NA	NA	0	1	1	1	NA	0	1	76.9
Anthierens, A. et al (2019)	0	NA	NA	NA	0	1	1	NA	NA	NA	NA	1	1	1	NA	0	1	66.7
Barreto, A. C. et al (2010)	1	1	1	1	0	1	1	NA	NA	NA	NA	1	1	1	NA	0	0	75.0
Beckham, S. G. & Earnest, C. P. (2000)	0	1	0	0	0	0	1	NA	NA	NA	NA	1	1	1	NA	0	1	50.0
Benito, P. J. et al (2016)	1	1	1	0	0	1	1	NA	NA	NA	NA	1	1	1	NA	1	0	75.0
Benito, P.; et al (2012)	0	1	1	0	0	1	1	NA	NA	NA	NA	1	1	1	NA	0	0	58.3
Berg, O. K. et al (2018)	1	NA	NA	NA	0	0	1	NA	NA	NA	NA	1	1	1	NA	1	0	66.7
Berthiaume, M. P. et al. (2015)	0	1	0	0	0	1	1	NA	NA	NA	NA	1	1	1	NA	0	0	50.0
Bertucci, W. M. et al (2015)	0	1	1	0	0	1	1	NA	NA	NA	1	1	1	1	NA	0	0	61.5

Binzen, C. A. et al (2001)	0	1	0	0	0	0	1	NA	NA	NA	NA	1	1	1	NA	0	1	50.0
Bloomer, R. J. (2005)	1	1	0	0	0	1	1	NA	NA	NA	NA	1	1	1	NA	0	0	58.3
Boudreaux, B. D. et al (2017)	1	0	0	NA	0	0	1	NA	NA	NA	NA	1	1	1	NA	0	0	45.5
Bradley, W. J. et al (2015)	0	NA	NA	NA	0	NA	1	NA	NA	NA	1	1	1	1	NA	1	NA	75.0
Brentano, M. A. et al (2016)	1	1	NA	0	0	0	1	NA	NA	NA	1	1	1	1	NA	1	0	66.7
Brewer, C. B. et al (2021)	1	0	0	NA	0	0	1	NA	NA	NA	NA	1	1	1	NA	0	0	45.5
Brisebois, M. F et al. (2022)	0	1	0	0	0	1	1	NA	NA	NA	NA	1	1	1	NA	0	0	50.0
Brunelli, D. T. et al (2019)	1	1	1	0	1	1	1	NA	NA	NA	1	1	1	1	NA	0	1	84.6
Cadieux, S. et al. (2014)	1	1	0	0	0	1	1	NA	NA	NA	1	1	1	1	NA	0	0	61.5
Caruso, J. F. et al. (2012)	0	1	0	0	0	0	1	NA	NA	NA	NA	1	1	1	NA	0	1	50.0
Caruso, J. F. et al. (2006)	0	0	0	NA	0	1	1	NA	NA	NA	NA	1	1	1	NA	1	1	63.6
Caruso, J. F. & Hernandez, D. A. (2002)	0	0	0	NA	0	0	1	NA	NA	NA	NA	1	0	1	NA	1	1	45.5
Caruso, J. F. et al. (2003)	0	1	1	0	1	1	1	NA	NA	NA	NA	1	1	1	NA	0	1	75.0
Caruso, J. F. et al. (2005)	0	0	0	NA	0	1	1	NA	NA	NA	NA	1	1	1	NA	0	1	54.5
Chatzinikolaou, A. et al. (2008)	1	NA	NA	NA	0	1	1	NA	NA	NA	NA	0	1	1	NA	0	0	55.6
Cherian, K. S. et al. (2018)	1	NA	NA	NA	1	NA	1	NA	NA	NA	NA	1	1	1	NA	0	NA	85.7
Correa, C. S. et al. (2015)	1	1	0	1	1	1	1	NA	0	NA	NA	1	1	1	NA	1	1	84.6
Correa, C. S. et al. (2014)	1	0	0	NA	0	1	1	NA	NA	NA	NA	1	1	1	NA	0	1	63.6
Costello, N. et al. (2019)	0	NA	NA	NA	0	NA	1	NA	NA	NA	1	1	1	1	NA	0	NA	62.5
Crommett, A. D. & Kinzey, S. J. (2004)	0	0	0	NA	0	1	1	NA	NA	NA	NA	1	1	1	NA	0	1	54.5
Cunha, F. A. et al. (2019)	1	NA	NA	NA	0	1	1	NA	NA	NA	NA	1	1	1	NA	0	1	77.8
Curtis, C. et al. (2023)	0	NA	NA	NA	0	1	1	NA	NA	NA	1	1	1	1	NA	1	NA	77.8
Da Silva, M. E. et al. (2007)	1	1	0	0	0	0	1	NA	NA	NA	NA	1	1	1	NA	1	1	66.7
Danoff, P. L. & Danoff, J. V. (1982)	0	0	0	NA	0	0	1	NA	NA	NA	NA	1	1	0	NA	0	0	27.3
De Groot, D. W. et al. (1998)	0	1	0	0	0	1	1	NA	NA	NA	NA	1	1	1	NA	0	1	58.3
Del-Cuerpo, I. et al. (2023)	1	1	0	0	0	1	1	0	0	NA	NA	1	1	1	NA	1	1	64.3
De Looze, M. P. et al. (1994)	0	1	0	0	0	1	1	NA	NA	NA	NA	1	1	1	NA	0	1	58.3
Dos Santos, T. R. et al. (2022)	1	1	1	1	0	1	1	NA	NA	NA	NA	1	1	1	NA	0	1	83.3
Drenowatz, C. et al. (2015)a	1	1	0	0	0	1	1	NA	0	NA	NA	1	1	1	NA	0	1	61.5
Drenowatz, C. et al. (2015)b	1	NA	NA	NA	0	NA	1	NA	NA	NA	NA	1	1	1	NA	1	NA	85.7
Dudley, G. A. et al. (1991)	0	NA	NA	NA	0	1	1	NA	NA	NA	NA	1	1	1	NA	0	1	66.7
Egger, T. & Flueck, J. L. (2020)	1	NA	NA	NA	0	1	1	NA	NA	NA	0	1	1	1	NA	0	NA	66.7
Falcone, P. H. et al. (2015)	1	0	0	NA	0	1	1	NA	NA	NA	NA	1	1	1	NA	1	1	72.7
Farinatti, P. T. & Castinheiras Neto, A. G. (2011)	1	1	1	0	0	1	1	NA	NA	NA	NA	1	1	1	NA	1	0	75.0
Farinatti, P. T. et al (2009)	1	0	1	NA	0	1	1	NA	NA	NA	NA	1	1	1	NA	0	0	63.6
Fatouros, I. G.; et al. (2005)	1	1	NA	0	0	1	1	NA	0	NA	1	1	1	1	NA	1	1	76.9
Ferrari, R. et al. (2018)	0	1	0	0	0	0	1	NA	NA	NA	NA	1	1	1	NA	0	1	50.0
Freitas, M. C. et al. (2019)	1	1	0	0	0	1	1	1	1	1	NA	1	1	1	NA	0	1	73.3
Garnacho-Castano, M. V. et al. (2019)	1	1	0	0	0	1	1	NA	NA	NA	NA	1	1	1	NA	1	0	66.7
Garnacho-Castano, M. V. et al. (2021)	1	NA	NA	NA	0	1	1	NA	NA	NA	NA	1	1	1	NA	0	0	66.7
Greer, B. K. et al. (2021)	1	1	0	0	1	1	1	NA	NA	NA	1	1	1	1	NA	0	1	76.9
Greer, B. K. et al. (2015)	0	0	NA	NA	0	1	1	NA	NA	NA	NA	1	1	1	NA	0	0	50.0
Gremeaux, V. et al. (2012)	1	NA	NA	NA	0	0	1	NA	0	NA	NA	1	1	1	NA	0	0	50.0
Guiraud, T. et al. (2015)	1	1	0	0	1	1	1	NA	NA	NA	NA	1	1	1	NA	1	1	83.3
Gutierrez, J. et al. (2015)	1	NA	NA	NA	0	1	1	NA	NA	NA	NA	1	1	1	NA	0	1	77.8
Haddock, B. L. & Wilkin, L. D. (2006)	1	1	1	0	0	1	1	NA	NA	NA	NA	1	1	1	NA	0	1	75.0

Hajj-Boutros, G. et al. (2022)	1	NA	NA	NA	0	1	1	NA	NA	NA	NA	1	1	1	NA	1	0	77.8
Haltom, R. W. et al. (1999)	0	1	0	0	1	1	1	NA	NA	NA	NA	1	1	1	NA	0	0	58.3
Harris, N. K. et al. (2017)	1	1	1	0	1	1	1	NA	NA	NA	1	1	1	1	NA	0	1	84.6
Harris, N. K. et al. (2016)	1	0	1	NA	0	1	1	NA	NA	NA	NA	1	1	1	NA	0	1	72.7
Harris, N. et al. (2018)	0	1	NA	NA	0	1	1	NA	NA	NA	NA	1	1	1	NA	0	1	70.0
Heden et al. (2017)	1	1	1	0	0	1	1	NA	NA	NA	1	1	1	1	NA	1	1	84.6
Hu, J. et al. (2021)	1	NA	NA	NA	1	0	1	NA	NA	NA	NA	1	1	1	NA	1	1	88.9
Hulsey, C. R. et al. (2012)	0	NA	NA	NA	0	0	1	NA	NA	NA	NA	1	1	1	NA	0	1	55.6
Hunter, G. R. et al. (2013)	0	1	NA	0	0	1	1	NA	0	NA	NA	1	1	1	NA	1	1	66.7
Hunter, G. R. et al. (2003)	1	1	0	0	0	1	1	NA	NA	NA	NA	1	1	1	NA	0	0	58.3
Jacobs, P. L. et al. (2002)	1	0	0	NA	0	1	1	NA	NA	NA	NA	1	1	NA	NA	0	1	60.0
Jarvinen, L. et al. (2022)	1	1	0	0	1	1	1	NA	NA	NA	0	1	1	1	NA	1	0	69.2
Joao, G. A. et al. (2021)	1	1	0	0	1	1	1	NA	NA	NA	NA	1	1	1	NA	0	1	75.0
Jun Liang, O. & Brownlee, I. A. (2017)	0	NA	NA	NA	0	NA	1	NA	NA	NA	1	1	1	1	NA	0	NA	62.5
Kalb, J. S. & Hunter, G. R. (1991)	0	0	0	NA	0	0	1	NA	NA	NA	NA	1	1	1	NA	0	0	36.4
Katch, F. I. et al. (1985)	0	NA	1	NA	0	0	1	NA	NA	NA	NA	1	1	1	NA	0	0	50.0
Keim, N. L. et al. (1996)	1	0	NA	NA	0	1	1	NA	0	NA	NA	1	1	1	NA	0	NA	60.0
Kelleher, A. R. et al. (2010)	0	NA	NA	NA	0	1	1	NA	NA	NA	NA	1	1	1	NA	0	0	55.6
Kemmler, W. et al. (2012)	1	1	0	0	1	1	1	1	NA	0	NA	1	1	1	NA	1	0	71.4
Kinnunen, H. et al. (2019)	1	NA	NA	NA	1	NA	1	NA	NA	NA	1	1	1	1	NA	1	NA	100.0
Koerhuis, C. L. et al (2003)	0	NA	NA	NA	0	1	1	NA	NA	NA	NA	1	1	1	NA	0	0	55.6
Lundstrom, E. A. et al. (2023)	1	NA	NA	NA	0	1	1	NA	NA	NA	1	1	1	1	NA	0	NA	77.8
Lyristakis, P. et al. (2019)	1	NA	NA	NA	0	1	1	NA	NA	NA	1	1	1	1	NA	1	1	90.0
Lytle, J. R. et al. (2019)	1	NA	NA	NA	0	1	1	NA	NA	NA	NA	1	1	1	NA	0	0	66.7
Magkos, F. et al. (2008)	0	1	0	0	1	1	1	NA	NA	NA	NA	1	1	1	NA	1	1	75.0
Magosso, R. F. et al. (2013)	1	1	0	0	0	1	1	NA	NA	NA	NA	1	1	1	NA	0	0	58.3
Marzolini, S. et al. (2012)	0	NA	NA	NA	0	1	1	NA	NA	NA	NA	1	1	1	NA	0	1	77.8
Mazzetti, S. A. et al. (2011)	0	1	1	0	0	1	1	NA	NA	NA	NA	1	1	1	NA	0	1	66.7
Mazzetti, S. et al. (2017)	0	1	1	0	1	1	1	NA	NA	NA	1	1	1	1	NA	0	1	76.9
Mazzetti, S. et al. (2011)	1	1	1	0	1	1	1	NA	NA	NA	NA	1	1	1	NA	0	1	75.0
McGuire, A. et al. (2023)	1	NA	NA	NA	0	1	1	NA	NA	NA	1	1	1	1	NA	0	NA	77.8
McMillan, D. W. et al. (2021)	1	1	1	0	1	1	1	NA	NA	NA	1	1	1	1	NA	1	1	92.3
Melanson, E. L. et al. (2005)	1	1	0	0	0	1	1	NA	NA	NA	NA	1	1	1	NA	1	0	66.7
Melanson, E. L. et al. (2002)	1	1	0	0	0	1	1	NA	NA	NA	NA	1	1	1	NA	1	1	75.0
Melby, C. et al. (1993)	1	0	1	NA	0	1	1	NA	NA	NA	NA	1	1	1	NA	1	0	72.7
Michalski, A. C. et al. (2023)	1	1	1	1	0	1	1	0	0	NA	NA	1	1	1	NA	0	1	71.4
Monteiro, A. G. et al. (2008)	1	0	0	NA	0	1	1	NA	NA	NA	NA	1	1	1	NA	0	0	54.5
Mookerjee, S. et al. (2016)	1	1	1	0	0	1	1	NA	NA	NA	NA	1	1	1	NA	0	1	75.0
Moon, J. M. et al. (2021)	0	NA	NA	NA	0	NA	1	NA	NA	NA	NA	1	1	1	NA	0	NA	71.4
Morgan, B. et al. (2003)	1	1	0	0	0	1	1	NA	NA	NA	NA	1	1	1	NA	0	0	50.0
Morris, C. E. et al. (2019)	0	NA	NA	NA	0	1	1	NA	NA	NA	NA	1	1	1	NA	1	1	88.9
Moss, S. L. et al. (2020)	0	NA	NA	NA	0	NA	1	NA	NA	NA	NA	1	1	1	NA	1	NA	71.4
Mukaimoto, T. & Ohno, M. (2012)	1	0	1	NA	0	1	1	NA	NA	NA	NA	1	1	1	NA	0	0	54.5
Munch, G. W. et al. (2018)	0	1	1	1	0	1	1	NA	0	NA	NA	1	1	1	NA	0	0	69.2
Nakagata, T. et al. (2018)	0	NA	NA	NA	1	1	1	NA	NA	NA	1	1	1	1	NA	0	0	70.0
Nakagata, T. et al. (2019)	0	1	0	NA	0	1	1	NA	NA	NA	NA	1	1	1	NA	0	0	54.5

Nakagata, T. et al. (2022)	1	1	0	0	0	1	1	NA	NA	NA	NA	1	1	1	NA	0	1	58.3
Nunez, T. P. et al. (2020)	1	0	1	NA	1	1	1	NA	NA	NA	NA	1	1	1	NA	0	1	81.8
Ortego, A. R. et al. (2009)	0	NA	NA	NA	0	1	1	NA	NA	NA	NA	1	1	1	NA	0	1	77.8
Paduan Joaquim, D. et al. (2018)	1	NA	NA	NA	0	0	1	NA	NA	NA	NA	1	1	1	NA	0	NA	50.0
Pafili, Z. K. et al. (2010)	0	1	0	0	0	1	1	NA	NA	NA	NA	1	1	1	NA	1	0	66.7
Parmar, P. et al. (2015)	0	1	0	0	1	1	1	NA	NA	NA	NA	1	1	1	NA	0	1	66.7
Petitt, D. S. et al. (2003)	0	1	0	0	0	1	1	NA	NA	NA	NA	1	1	1	NA	1	0	58.3
Phillips, W. T. & Ziuraitis, J. R. (2003)	0	NA	NA	NA	0	1	1	NA	NA	NA	NA	0	1	1	NA	0	1	55.6
Phillips, W. T. & Ziuraitis, J. R. (2004)	1	NA	NA	NA	0	1	1	NA	NA	NA	NA	0	1	0	NA	1	0	44.4
Quintero, A. P. et al. (2018)	1	1	0	0	0	0	1	NA	NA	NA	NA	1	1	1	NA	1	1	66.7
Rangan, V. V. et al. (2011)	1	1	0	0	0	NA	1	NA	0	NA	1	1	1	1	NA	0	NA	58.3
Ratamess, N. A. et al. (2016)	1	1	1	0	0	1	1	1	1	0	NA	1	1	1	NA	1	1	80.0
Ratamess, N. A. et al. (2007)	0	1	1	0	0	1	1	NA	NA	NA	NA	1	1	1	NA	0	0	66.7
Ratamess, N. A. et al. (2018)	1	1	0	0	0	1	1	NA	NA	NA	NA	1	1	1	NA	1	1	66.7
Ratamess, N. A. et al. (2014)	0	1	1	0	0	1	1	NA	NA	NA	NA	1	1	1	NA	0	1	75.0
Ratamess, N. A. et al. (2015)	1	0	0	NA	0	1	1	NA	NA	NA	NA	1	1	1	NA	1	1	63.6
Rawson, E. S. & Walsh, T. M. (2010)	1	NA	NA	NA	0	1	1	NA	NA	NA	NA	1	1	1	NA	0	0	66.7
Realzola, R. A. et al. (2021)	1	NA	NA	NA	0	1	1	NA	NA	NA	NA	1	1	1	NA	0	0	66.7
Reed, J. L. et al (2013)	0	NA	NA	NA	1	NA	1	NA	NA	NA	1	1	1	1	NA	0	NA	87.5
Reed, J. L. et al (2014)	1	NA	NA	NA	0	NA	1	NA	NA	NA	1	1	1	1	NA	0	NA	62.5
Reeve, M. D. et al. (2014)	1	NA	NA	NA	0	1	1	NA	NA	NA	1	1	1	1	NA	1	1	90.0
Reis, V. M. et al. (2017)	1	1	0	0	1	1	1	NA	NA	NA	0	1	1	1	NA	0	0	61.5
Reis, V. M. et al. (2019)	0	1	0	0	0	1	1	NA	NA	NA	NA	1	1	1	NA	0	0	58.3
Robergs, R. A. et al. (2007)	0	1	0	0	0	1	1	NA	NA	NA	NA	1	0	1	NA	0	0	41.7
Roberson, K. B. et al. (2017)	1	1	1	0	0	1	1	NA	NA	NA	NA	1	1	1	NA	0	1	66.7
Rustaden, A. M. et al. (2020)	0	NA	NA	NA	0	1	1	NA	NA	NA	NA	1	1	1	NA	0	0	66.7
Salagaras, B. S. et al. (2021)a	0	NA	NA	NA	0	NA	1	NA	NA	NA	1	1	1	1	NA	1	NA	75.0
Salagaras, B. S. et al. (2021)b	1	NA	NA	NA	0	1	1	NA	NA	NA	1	1	1	1	NA	1	NA	77.8
Santa-Clara, H. et al. (2018)	1	NA	NA	NA	0	1	1	NA	NA	NA	1	1	1	1	NA	0	0	70.0
Santos, D. A. et al. (2014)	1	NA	NA	NA	0	1	1	NA	NA	NA	1	1	1	1	NA	1	NA	88.9
Sarafian, D. et al. (2016)	1	NA	NA	NA	0	1	1	NA	NA	NA	NA	1	1	1	NA	1	0	77.8
Schaal, K. et al. (2021)	1	NA	NA	NA	1	1	1	NA	NA	NA	1	1	1	1	NA	0	1	81.8
Scheffer, J. H. et al. (2023)	1	NA	NA	NA	0	1	1	NA	NA	NA	NA	1	1	1	NA	0	NA	75.0
Scott, C. B. (2006)	0	1	0	0	0	1	1	NA	NA	NA	0	1	1	1	NA	0	0	53.8
Scott, C. B. (2012)	0	1	0	0	0	1	1	NA	NA	NA	NA	1	1	1	NA	0	0	50.0
Scott, C. B. et al. (2009)	0	1	0	0	0	1	1	NA	NA	NA	NA	1	1	1	NA	0	0	50.0
Scott, C. B. et al. (2011)a	0	1	0	0	0	1	1	NA	NA	NA	NA	1	1	1	NA	0	0	50.0
Scott, C. B. et al. (2011)b	0	1	0	0	1	1	1	NA	NA	NA	NA	1	1	1	NA	0	0	58.3
Scott, J. M. et al. (2014)	0	NA	NA	NA	0	1	1	NA	NA	NA	NA	1	1	1	NA	0	1	66.7
Seliger, V. et al. (1980)	0	NA	NA	NA	0	0	1	NA	NA	NA	NA	0	1	1	NA	0	1	44.4
Seliger, V. et al. (1968)	1	0	NA	NA	0	0	1	NA	0	NA	NA	0	1	1	NA	0	0	27.3
Shannon, K. A. et al. (2005)	1	1	0	1	1	1	1	NA	0	NA	NA	1	1	1	NA	1	0	76.9
Smith L. E. et al. (2022)	1	NA	NA	NA	1	1	1	NA	NA	NA	NA	1	1	1	NA	0	1	88.9
Stec, M. J. & Rawson, E. S. (2012)	1	NA	NA	NA	0	1	1	NA	NA	NA	NA	1	1	1	NA	0	1	77.8
Steele, J. et al. (2018)	1	1	1	0	0	0	1	NA	NA	NA	NA	1	1	1	NA	0	0	58.3
Taylor, H. L. et al. (2022)	1	NA	NA	NA	0	NA	1	NA	NA	NA	NA	1	1	1	NA	0	NA	71.4

Thornton, M. K. & Potteiger, J. A. (2002)	1	1	1	0	0	1	1	NA	NA	NA	NA	1	1	1	NA	0	0	66.7
Thornton, M. K. et al. (2011)	1	1	1	0	0	1	1	NA	NA	NA	NA	1	1	1	NA	0	0	66.7
Tornberg, A. B.; et al. (2017)	1	NA	NA	NA	0	1	1	NA	NA	NA	NA	1	1	1	NA	0	1	77.8
Traversa, C. et al. (2022)	1	NA	NA	NA	0	1	1	NA	NA	NA	NA	1	1	1	NA	0	NA	75.0
Trexler, E. T. et al. (2020)	1	1	1	1	0	1	1	1	1	1	1	1	1	1	NA	1	1	93.8
Trexler, E. T. et al. (2019)	1	1	1	1	1	1	1	1	1	1	1	1	1	1	NA	1	1	100.0
Van Etten, L. M et al. (1997)	0	0	NA	NA	0	1	1	NA	0	NA	NA	1	1	1	NA	0	NA	50.0
van Etten, L. M. et al. (1996)	0	NA	NA	NA	0	1	1	NA	NA	NA	NA	1	1	1	NA	1	1	77.8
Vandenbrink, D. N. et al. (2018)	1	NA	NA	NA	0	1	1	NA	NA	NA	NA	1	1	1	NA	0	1	77.8
Vezina, J. W. et al. (2014)	1	1	0	0	0	1	1	NA	NA	NA	NA	1	1	1	NA	1	1	75.0
Vincent, H. K. et al. (2014)	0	1	1	0	0	1	1	NA	NA	NA	NA	1	1	1	NA	0	1	66.7
Walker, E. J. et al. (2016)	0	NA	NA	NA	0	1	1	NA	NA	NA	1	1	1	1	NA	1	NA	77.8
Wickham, J. B. et al. (2017)	0	1	1	1	1	1	1	NA	NA	NA	NA	1	1	1	NA	0	0	75.0
Williams, B. M. & Kraemer, R. R. (2015)	1	1	1	0	0	1	1	NA	NA	NA	NA	1	1	1	NA	0	1	75.0
Wilmore, J. H. et al. (1978)	0	NA	NA	NA	0	1	1	NA	NA	NA	NA	0	1	1	NA	0	0	44.4
Yoshida, A. et al. (2019)	0	NA	NA	NA	0	1	1	NA	NA	NA	NA	1	1	1	NA	1	NA	75.0
Zanders, B. R. et al. (2021)	1	NA	NA	NA	0	1	1	NA	NA	NA	1	1	1	1	NA	1	NA	88.9
Zotou, E. et al. (2010)	1	1	0	0	0	1	1	NA	NA	NA	NA	1	1	1	NA	0	1	66.7
